# Synthesis and Biological Evaluation of New Pleuromutilin Derivatives as Antibacterial Agents

**DOI:** 10.3390/molecules191119050

**Published:** 2014-11-19

**Authors:** Ruo-Feng Shang, Guan-Hua Wang, Xi-Ming Xu, Si-Jie Liu, Chao Zhang, Yun-Peng Yi, Jian-Ping Liang, Yu Liu

**Affiliations:** 1 Key Laboratory of New Animal Drug Project of Gansu Province, Key Laboratory of Veterinary Pharmaceutical Development, Lanzhou Institute of Husbandry and Pharmaceutical Sciences of CAAS, Ministry of Agriculture, Lanzhou 730050, China; E-Mails: shangrf1974@163.com (R.-F.S.); YJL.BZN@163.com (C.Z.); yiyp@foxmail.com (Y.-P.Y.); 2 School of Public Health, Lanzhou University, Lanzhou 730000, China; E-Mail: wgh2650@163.com; 3 Department of Toxicology, School of Public Health, Guiyang Medical University, Guiyang 550004, China; E-Mail: xmngxu@gmail.com; 4 College of Chemical Engineering, Shijiazhuang University, Shijiazhuang 050035, China; E-Mail: liusijie1982@163.com

**Keywords:** pleuromutilin derivatives, antibacterial activity, synthesis, molecular docking, ADMET properties

## Abstract

Several pleuromutilin derivatives possessing thiadiazole moieties were synthesized via acylation reactions under mild conditions. The *in vitro* antibacterial activities of the derivatives against methicillin-resistant *S. aureus*, methicillin-resistant *S. epidermidis*, *S. aureus*, *S. epidermidis*, *E. coli*, and *B. cereus* were tested by the agar dilution method and Oxford cup assay. All the screened compounds displayed potent activity. Compound **6d** was the most active antibacterial agent because of its lowest MIC value and largest inhibition zone. Docking experiments were performed to understand the possible mode of the interactions between the derivatives and 50S ribosomal subunit. Moreover, the absorption, distribution, metabolism, excretion and toxicity properties of the synthesized compounds were analyzed after prediction using the Advanced Chemistry Development/Percepta Platform available online.

## 1. Introduction

**Figure 1:**
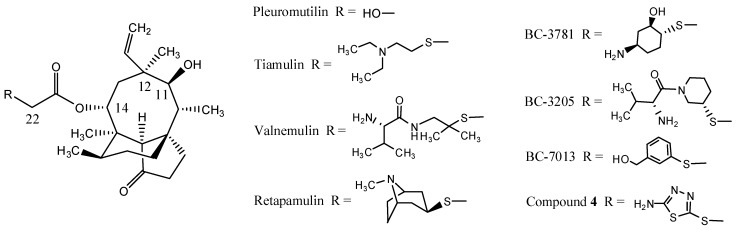
Structural formulas of pleuromutilin, tiamulin, valnemulin, retapamulin, BC-3781, BC-3205 and BC-7013.

The natural compound pleuromutilin (Figure 1) was first discovered and isolated in a crystalline form from *Pleurotus mutilus* and *P. passeckerianus* in 1951 [1]. Pleuromutilin is a diterpene, constituted of a rather rigid 5-6-8 tricyclic carbon skeleton with eight stereogenic centers [2,3] and a glycolic acid chain at C-14 which was considered as the main molecular modification point [4]. Although pleuromutilin has a modest antibacterial activity, the modifications of the C-14 position have led to three drugs: tiamulin, valnemulin, and retapamulin (Figure 1). Tiamulin and valnemulin are used in veterinary medicine for pigs and poultry. Retapamulin was approved as a topical antimicrobial agent for the treatment of human skin infections in 2007 by FDA [5,6]. Extensive efforts were made to formulate BC-3781, BC-3205 and BC-7013 (Figure 1) for human use [7,8] after the success of retapamulin.

The interaction modes that pleuromutilin derivatives selectively inhibit bacterial proteins synthesis were demonstrated as early as 1974 [9]. Crystallography data, utilizing a structure of 50S ribosomal subunit from *Deinococcus radiodurans* in complex with tiamulin, demonstrated that the interactions of tricyclic core of the tiamulin are mediated through hydrophobic interactions and hydrogen bonds, which are formed mainly by the nucleotides of the domain V of 23S rRNA at the peptidyl transferase center (PTC) [10,11]. The C-11 hydroxyl group and the C-21 keto group of pleuromutilin derivatives are located in a position suitable for hydrogen bonding to G-2505 phosphate and G-2061, respectively, while the C-14 side chain prevents the peptidyl transferase rRNA bases U-2584 and U-2585 from tRNA binding to the P-site [11]. This effect is of particular importance as Long *et al.* suggestion that the nature of the pleuromutilin side chain is potentially critical for overcoming resistance mediated by the mutation of the ribosomal protein [9,12].

Compounds containing a 1,3,4-thiadiazole moieties exhibit a wide range of biological activities such as antibacterial [13], antitumor [14] and antifungal activities [15]. Recently, we described a series of pleuromutilin derivatives with potent antibacterial activities from a 1,3,4-thiadiazole-based lead, 14-O-[(2-amino-1,3,4-thiadiazol-5-yl) thioacetyl] mutilin (**4**, Figure 1). Two compounds with anilino substituents in their side chains showed stronger antibacterial activities [16]. However, they also showed low chemical stabilities in the subsequent research because the amino group on the phenyl ring is easily oxidized in the air. To resolve this problem, the amino or phenyl ring was replaced by other substituents and some new pleuromutilin derivatives bearing 1,3,4-thiadiazoles were designed and synthesized. Moreover, detailed antibacterial activities and molecular docking studies of all synthesized compounds were performed to explore their binding models with 50S ribosomal subunit.

## 2. Results and Discussion

### 2.1. Chemistry

**Scheme 1:**
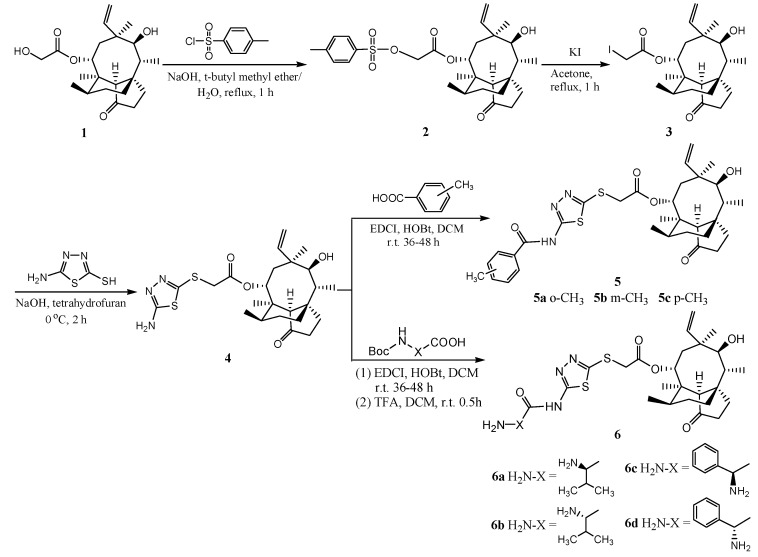
Synthesis of compounds **5a**–**c** and **6a**–**d**.

Pleuromutilin derivatives **5a**–**c** and **6a**–**d** were synthesised from the lead compound 14-O-[(2-amino-1,3,4-thiadiazol-5-yl) thioacetyl] mutilin (**4**) which was synthesized in turn from 22-O-tosylpleuromutilin (**2**) according to previous literature [4,16]. Compounds **5a**–**c** and **6a**–**d** were directly obtained by condensation reactions between the amino group of compound **4** and the carboxyl group of methyl-substituted aryl carboxylic acids or amino acids which amino groups were protected by *tert*-butoxycarbonyl (BOC) groups. The reactions were performed at room temperature in the presence of 1-ethyl-3-(3-dimethyllaminopropyl) carbodiimide hydrochloride (EDCI) and 1-hydroxybenzotriazole (HOBt) which was used to suppress racemization and improve the efficiency of the amide synthesis (Scheme 1). The protected amino groups were hydrolyzed with TFA for 30 min and the compounds **6a**–**d** were obtained. The synthesis and the IR, ^1^H-NMR, ^13^C-NMR and HRMS spectra of all the new compounds were reported in the Supplementary data.

### 2.2. Antibacterial Activity

The synthesized pleuromutilin derivatives **5a**–**c** and **6a**–**d** along with tiamulin used as reference drug were screened for their *in vitro* antibacterial activity against MRSA, MRSE, *S. aureus*, *S. epidermidis*, *E. coli*, and *B. cereus.* The antibacterial activities were reported in Table 1 as the minimum inhibitory concentration (MIC) using the agar dilution method and in Table 2 as the zones of inhibition using Oxford cup assay.

**Table 1:** Antibacterial activity (MIC) of the synthesized pleuromutilin derivatives.

Comp.	MIC (μg/mL)					
	MRSA	MRSE	*S. aureus*	*S. epidermidis*	*E. coli*	*B. cereus*
**5a**	32	64	32	32	≥128	16
**5b**	64	≥128	64	64	≥128	32
**5c**	32	32	16	32	64	4
**6a**	8	16	4	16	32	2
**6b**	32	32	32	32	64	16
**6c**	8	32	8	16	64	8
**6d**	0.5	4	0.5	2	32	0.25
Tiamulin	0.5	2	0.5	2	16	0.25

**Table 2:** Zone of Inhibition for MRSA, MRSE, *S. aureus*, *S. epidermidis*, *E. coli* and *B. cereus* in mm.

Comp.	MRSA		MRSE		*S. aureus*		*S. epidermidis*		*E. col*		*B. cereus*	
	320	160	320	160	320	160	320	160	320	160	320	160
**5a**	16.51	13.22	16.40	12.93	16.23	13.39	16.46	13.82	13.16	11.65	17.08	15.10
**5b**	14.03	11.67	13.88	12.34	13.42	11.14	14.02	12.53	12.97	11.13	15.51	13.55
**5c**	16.47	13.14	16.85	13.46	17.35	13.89	17.05	13.66	12.23	10.83	18.73	15.26
**6a**	17.51	14.49	17.35	13.89	18.82	14.75	18.31	14.32	14.22	12.17	19.33	16.51
**6b**	16.05	12.84	15.69	12.30	15.93	12.04	16.17	12.12	12.59	10.86	17.42	14.05
**6c**	17.11	14.28	15.13	12.21	17.44	14.39	16.83	13.25	14.85	12.67	18.25	13.91
**6d**	19.46	17.62	17.28	15.03	22.67	18.82	19.15	16.73	16.22	13.61	23.84	19.39
Tiamulin	20.35	17.84	17.93	15.75	22.23	19.04	20.58	16.05	17.84	15.29	23.18	20.57

All of screened compounds showed potent activity against all the strains except *E. coli*, and slightly less potent against resistant strains (MRSA and MRSE) than standard ones (*S. aureus* and *S. epidermidis*). In particular, compound **6d** bearing the l-(‒)-phenylglycinamide group on the C-14 glycolic acid side chain, was found to have the most potent activities against MRSA (MIC = 0.5 μg/mL), MRSE (MIC = 4 μg/mL), *S. aureus* (MIC = 0.5 μg/mL), *S. epidermidis* (MIC = 2 μg/mL) and *B. cereus* (MIC = 0.25 μg/mL) comparable to tiamulin. However its stereoisomer, compound **6c** showed moderate activities against MRSA, MRSE, *S. aureus*, *S. epidermidis* and *B. cereus*.

The results of Oxford cup assay correspond with that obtained by agar dilution method (MIC) as a whole. Compound **6d** showed the best growth inhibition against the pathogens particularly MRSA, MRSE and *B. cereus* comparable to tiamulin. Compounds **6a** and **6c** showed moderate growth of MRSA, MRSE, *S. aureus*, *S. epidermidis* and *B. cereus*. However, all the compounds exhibited weak activity against the *E. coli*.

Compounds **6a**–**d** with an amino group on the terminal C-14 glycolic acid side chain presented improved activity against all the strains except *E.coli* compared with the compounds **5a**–**c** bearing the methylbenzene group on the terminal C-14 glycolic acid side chain. The results of antibacterial activities indicated that the introduction of the amino group into the C-14 glycolic acid side chain could enhance antibacterial activity, which was consistent with previous reported [4,16].

Among the above mentioned compounds, **6d** was found inhibit the growth of the organisms at minimum concentration and found to possess good antibacterial activity. The MIC values and the observed growth inhibition have demonstrated that **6d** might act as potent antibacterial agent.

### 2.3. Molecular Docking Study

**Figure 2:**
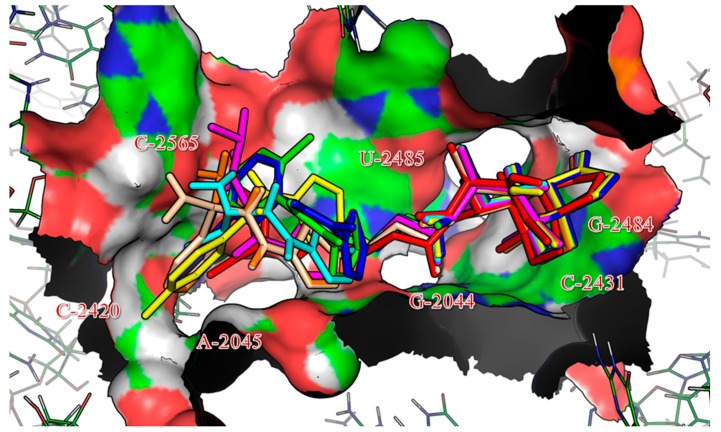
Superimposition of native ligand, tiamulin (colored by red) and the best conformations of **5a** (green), **5b** (blue), **5c** (yellow), **6a** (magenta), **6b** (cyan), **6c** (orange) and **6d** (wheat) docked to the binding pocket of ribosome (1XBP).

To investigate the binding mode of the series of pleuromutilin derivatives, docking simulations were performed with Homdock software [17]. The re-docking of tiamulin **2** into 1XBP^12^ placed the drug in the same conformation as that in the X-ray structure (RMSD 0.7 Å). The docking results for the seven compounds revealed a similar binding pattern within the binding site, with a RMSD range of 1.00 to 1.23 Å, as documented for tiamulin and presented in Figure 2 which shows a superimposition of tiamulin and the seven docked compounds. Furthermore, the docking results are in good agreement with the interactions highlighted in the crystal structure of 50S ribosomal subunit from *Deinococcus radiodurans* in complex with tiamulin.

**Figure 3:**
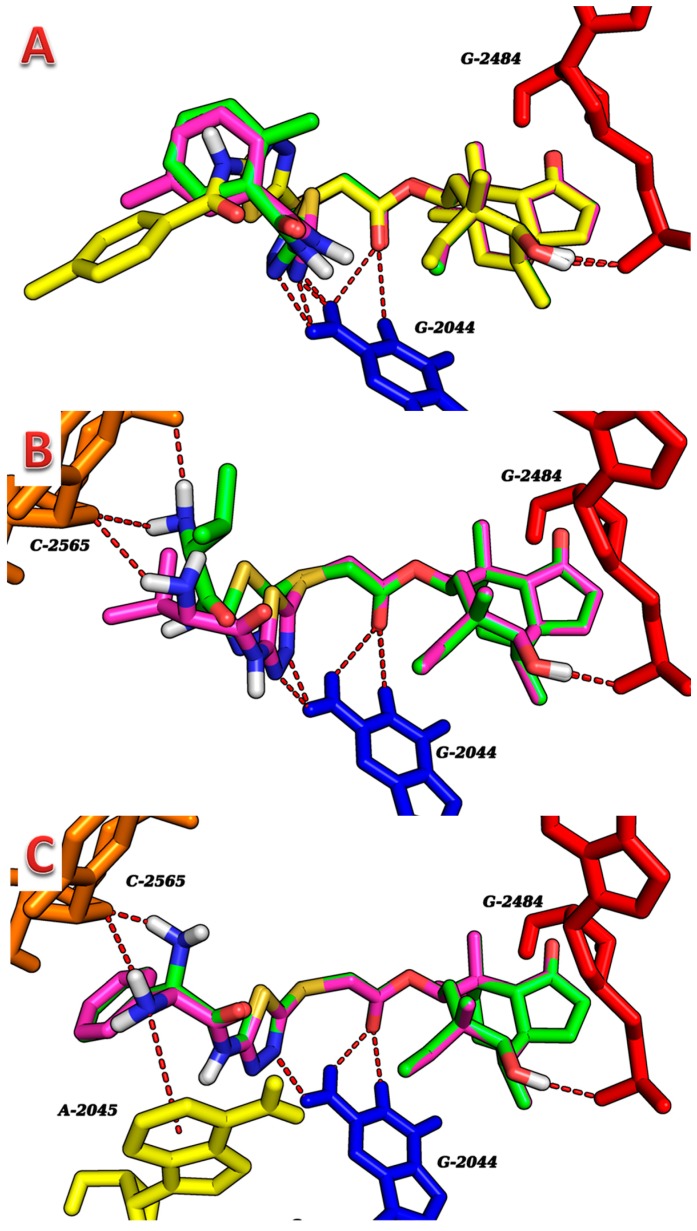
Docking modes of the synthesized compounds into 1XBP. (**A**) **5a** (green), **5b** (magenta) and **5c** (yellow); (**B**) **6a** (green) and **6b** (magenta) to 1XBP; (**C**) **6c** (green) and **6d** (magenta). Important residues are drawn in stick and different color. Hydrogen bonds and cation–π interaction are showed as dashed red lines.

The docking simulations revealed hydrogen bonding played the most important role in the binding of the compounds to 1XBP (Table 3; Figure 3). However, other interactions, such as hydrophobic ones are not shown in Table 3 or Figure 3. The seven compounds exhibited multiple binding modes into 1XBP with the binding free energies (ΔG_b_) in the range of −10.42 to −15.09 kcal/mol.

**Table 3:** Binding free energy, number of noncovalent molecular interactions and RMSD.

Compound	ΔG_b_ (kcal/mol)	Noncovalent Molecular Interaction				RMSD(Å)
		Hydro I Interaction	Atom of Compound	Residue	Distance (Å)	
**5a**	−10.42	H-bonding	OH (8-membered ring)	G-2484	2.0	1.21
		H-bonding	N (thiadiazole)	G-2044	2.4	
		H-bonding	N (thiadiazole)	G-2044	2.7	
**5b**	−10.56	H-bonding	OH (8-membered ring)	G-2484	2.0	1.23
		H-bonding	N (thiadiazole)	C-2044	2.1	
		H-bonding	N (thiadiazole)	G-2044	2.2	
**5c**	−11.50	H-bonding	OH (8-membered ring)	G-2484	2.1	1.08
		H-bonding	C=O (ester)	G-2044	2.7	
		H-bonding	C=O (ester)	G-2044	2.8	
**6a**	−12.21	H-bonding	OH (8-membered ring)	G-2484	1.9	1.00
		H-bonding	C=O (ester)	G-2044	2.5	
		H-bonding	C=O (ester)	G-2044	2.5	
		H-bonding	NH_2_ (terminal)	C-2565	2.1	
		H-bonding	NH_2_ (terminal)	C-2565	2.7	
**6b**	−11.69	H-bonding	OH (8-membered ring)	G-2484	1.9	1.00
		H-bonding	C=O (ester)	G-2044	2.5	
		H-bonding	C=O (ester)	G-2044	2.5	
		H-bonding	N (thiadiazole)	G-2044	2.1	
		H-bonding	N (thiadiazole)	G-2044	2.3	
		H-bonding	NH_2_ (terminal)	C-2565	2.6	
**6c**	−14.14	H-bonding	OH (8-membered ring)	G-2484	2.1	1.02
		H-bonding	C=O (ester)	G-2044	2.3	
		H-bonding	C=O (ester)	G-2044	2.3	
		H-bonding	N (thiadiazole)	G-2044	2.3	
		H-bonding	NH_2_ (phenylglycinamide)	C-2565	1.6	
**6d**	−15.09	H-bonding	OH (8-membered ring)	G-2484	2.1	1.02
		H-bonding	C=O (ester)	G-2044	2.3	
		H-bonding	C=O (ester)	G-2044	2.3	
		H-bonding	N (thiadiazole)	G-2044	2.2	
		H-bonding	NH_2_ (phenylglycinamide)	C-2565	2.3	
		Cation–π interaction	N (pyrrolidine)	G-2045	3.7	

Compounds **5a** and **5b** exhibited the similar docking mode with three hydrogen bonds between their OH (8-membered ring) and N (thiadiazole) with G-2484 and G-2044 (Table 3; Figure 3A). Compound **5c** showed a different docking mode, it exhibited two hydrogen bonds between its C=O (ester) group with G-2044 except one hydrogen bond between its OH (8-membered ring) with G-2484. The higher binding affinity of **5c** compared to **5a** and **5b** may be explained by the terminal methylbenzene group protruding into a hydrophobic area formed by resides of A-2420, C-2046 and A-2045. Although compound **6a** is a stereoisomer of **6b**, it showed a slight different docking mode compared to **6b** (Table 3; Figure 3B). Compounds **6c** and **6d** adopted very similar conformations and hydrogen bonds interactions except for terminal phenylglycinamide group. However, compound **6d** displayed a higher binding affinity (ΔG_b_ = −15.09) than **6c**, perhaps because of a cation-π interaction formed between the N (pyrrolidine) and G-2045 (Table 3; Figure 3C). Because the difference of the binding free energies caused by different binding modes for the stereoisomers, their showed significant difference in antibacterial activities.

**Figure 4:**
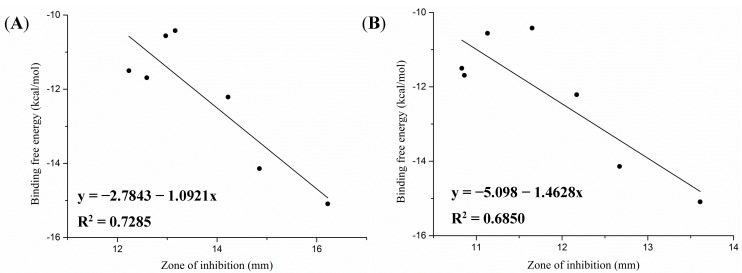
Correlation between binding free energy (ΔGb, kcal/mol) and antibacterial activity (zone of inhibition; mm) against *E. coli*. for scereened compounds with 320 μg/mL (**A**) and 160 μg/mL (**B**).

The molecular docking results revealed a rational correlation between the predicted binding affinities (binding free energies) and the antibacterial activity. Because X-ray structures of the 50S ribosomal subunits are available only for *E. coli* (PDB ID: 2AW4), we investigated the linear relationships between the binding free energy (ΔGb, kcal/mol) and the zone of inhibition (mm) for 320 and 160 μg/mL concentrations of *E. coli* based on the high similarity in domain V of 23S rRNA at the PTC between 1XBP and 2AW4 [16]. The results revealed a direct reasonable correlation between the binding free energy and the zone of inhibition, with correlation coefficients (R2) of 0.6626 and 0.7658 for the screened compounds as illustrated in Figure 4.

### 2.4. Prediction of ADMET Properties

The prediction of absorption, distribution, metabolism, excretion and toxicity properties (ADMET) facilitates the lead compound discovery process, which is crucial for reducing ADMET problems in the later stage by optimization of those properties during the early stages of drug discovery. The ADMET properties of the synthesized compounds were predicted, including absorption, extent of blood-brain barrier permeation (Log BB), rate of passive diffusion/permeability (Log PS), plasma protein binding (PPB), volume of distribution (Vd) and median lethal dose (LD_50_), as well as acid dissociation constant (pKa) and the value of the octanol-water partitioning coefficient as the logarithmic ratio (Log *P*) using the Advanced Chemistry Development (ACD)/Percepta Platform available online.

According to the values shown in Table 4, the compounds with similar side chains had the same ADMET properties because of their similar pKa and Log *p* values predicted by ACD/Lab. It is well known that the log *p* value can be used to assess the hydrophilicity of a compound. A high log *p* value is associated with poor absorption or permeation, and the ideal value should be below about 5 [18]. Our study suggested that compounds **6a**–**d** had log *p* values of 3.88–4.64, while **5a**–**c** were slightly above this limit. Compounds **5a**–**c** can be more efficiently absorbed in the human intestine with the highest jejunal permeability coefficient (*P*_eff_) at pH 6.5 than the compounds **6a**–**b**. Log PS and Log BB are main parameters characterizing different aspects of the analyzed process when the blood-brain barrier (BBB)-transport potential of drugs or other molecules is measured experimentally [19]. The higher lipophilicity and lower protonation state made compounds **5a**–**c** permeate the membranes of endothelial cells more easily (Log BB = 0.29; Log PS = −1.7). PPB values represent the overall fraction of drug bound in human plasma such as albumin, α-1-acid glycoprotein, lipoproteins, and transcortin. All of the compounds were likely to be bound to serum proteins with a high degree of combination (PPB > 95%) which led to their lower volume of distribution (Vd < 5 L/kg). All of the compounds were mildly toxic with LD_50_ in the range of 730–1,400 mg/kg predicted for mouse after oral administration.

**Table 4:** Prediction ADMET properties for examined pleuromutilin derivatives.

Comp.	ADMET Parameter						pKa		c Log *p*
	*P*_eff_ (cm/s) ^a^	Log BB ^b^	Log PS ^c^	PPB (%) ^d^	Vd (L/kg) ^e^	LD_50_ (mg/kg) ^f^	Acid	Base	
**5a**	6.39 × 10^−4^	0.29	−1.7	99.27	4.15	1400	10.40	-	6.29
**5b**	6.39 × 10^−4^	0.29	−1.7	99.27	4.15	1400	10.40	-	6.29
**5c**	6.39 × 10^−4^	0.29	−1.7	99.27	4.15	1400	10.40	-	6.29
**6a**	5.12 × 10^−4^	0.02	−2.3	97.14	3.15	730	10.30	7.70	3.88
**6b**	5.12 × 10^−4^	0.02	−2.3	97.14	3.15	730	10.30	7.70	3.88
**6c**	5.41 × 10^−4^	0.2	−2.4	98.44	1.60	970	7.80	11.80	4.64
**6d**	5.41 × 10^−4^	0.2	−2.4	98.44	1.60	970	7.80	11.80	4.64

^a^ Jejunal permeability coefficients at pH 6.5 that depends on paracellular and transcellural transport routes and unstirred water layer resistance; ^b^ Extent of brain penetration determined by ratio of total drug concentrations in tissue and plasma at steady-state conditions; ^c^ Rate of brain penetration. PS stands for Permeability-Surface area product and is defined from the kinetic equation of capillary transport; ^d^ The cumulative percentage of a compound bound to human plasma proteins, such as albumin, alpha_1_-acid glycoprotein and others (RI ≥ 0.30); ^e^ Prediction (probably 90%) of Volume of Distribution (Vd) regarding the effect of physicochemical properties (Log *p* and ionization) on drug distribution in the body; ^f^ Acute toxicity (LD_50_) for mouse after oral administration (RI ≥ 0.46).

As we know, toluene group noticeably increased the lipophilicity compare to amino group, while the compound lipophilicity can more or less affect their ADMET properties. Compounds **5a**–**c** with toluene group to the side chain showed slightly better ADMET properties than compounds **6a**–**d**. However, compound **6d** may serve as a possible drug-like compound by comprehensive considering its antibacterial activity and ADMET properties.

Based on the structural fragment and atomic contributions which are associated with data drawn from quantitative structure activity relationship (QSAR) studies, the predictions for ADMET properties using ACD/Percepta Platform can be efficiently evaluated *in silico* approaches, thereby accelerating the drug discovery process [20].

## 3. Experimental Section

### 3.1. General

All reagents and solvents were of analytical grade and used without further purification. All reactions were monitored by TLC on 0.2-mm-thick silica gel GF254 pre-coated plates. After elution, the plates were visualized under UV illumination at 254 nm for UV active materials. Further visualization was achieved by staining with a 0.05% KMnO4 aqueous solution. All column chromatography purifications were carried out on silica gel (200–300 mesh, Qingdao Haiyang Chemical Co., Ltd, Qingdao, China) with conventional methods. The melting points of the synthesized compounds were determined on a Tianda Tianfa YRT-3 apparatus (Tianjin, China) with open capillary tubes and were uncorrected. IR spectra were obtained on a NEXUS-670 spectrometer (Nicolet Thermo, Edina, MN, USA) using KBr thin films and the absorptions are reported in cm^−1^. NMR spectra were recorded in appropriate solvents using a Bruker-400 MHz spectrometer (Bruker BioSpin, Zürich, Zürich State, Switzerland). The chemical shifts (δ) were expressed in parts per million (ppm) relative to tetramethylsilane. The multiplicities of the NMR signals were designated as s (singlet), d (doublet), t (triplet), q (quartet), m (multiplet), br (broad), *etc*. High-resolution mass spectra (HRMS) were obtained on a Bruker Daltonics APEX II 47e mass spectrometer equipped with an electrospray ion source.

### 3.2. Synthesis

#### 3.2.1. General Procedure for the Synthesis of Compounds **5a**–**c**

A mixture of methyl-substituted benzoic acid (2.2 mmol), compound **4** (0.98 g, 2 mmol), 1-ethyl-3-(3-dimethylaminopropyl) carbodiimide hydrochloride (0.42 g, 2.2 mmol), 1-hydroxy-benzotriazole (0.30 g, 2.2 mmol) and dichloromethane (50 mL) was stirred at room temperature for 28 h. The mixture was washed with saturated aqueous NaHCO_3_ and water, dried with anhydrous MgSO_4_ overnight and rotary evaporated to dryness. The crude residue thus obtained was purified by silica gel column chromatography (petroleum ether-ethyl acetate 2:1 v/v) to afford the pure desired compounds **5a**–**5c**.

*14-O-[(2-Methylbenzamide-1,3,4-thiadiazol-5-yl) thioacetyl] mutilin* (**5a**): Compound **5a** was prepared according to the general procedure from 14-*O*-[(2-amino-1,3,4-thiadiazol-5-yl)thioacetyl] mutilin (**4**) and *o*-methylbenzoic acid. The crude product was purified by silica gel column chromatography to give 0.64 g (yield 52%) of compound **5a**, mp 113–115 °C. IR (KBr): υ_max_ cm^−1^ 3448, 2931, 1733, 1676, 1533, 1458, 1302, 1151, 1116, 1049, 893, 737, 666 cm^−1^. ^1^H-NMR (CDCl_3_) δ ppm 7.74 (d, *J* = 7.3 Hz, 1H), 7.48–7.42 (m, 1H), 7.34 (s, 2H), 6.41 (q, *J* = 17.4, 11.0 Hz, 1H), 5.76 (d, *J* = 8.4 Hz, 1H), 5.30 (q, *J* = 11.0, 1.1 Hz, 1H), 5.15 (q, *J* = 17.4, 1.2 Hz, 1H), 3.99–3.88 (t, 2H), 3.33 (d, *J* = 6.3 Hz, 1H), 2.53 (s, 3H), 2.33–2.12 (m, 3H), 2.08–2.01 (m, 2H), 1.75 (q, *J* = 14.4, 2.4 Hz, 1H), 1.62 (m, *J* = 17.5, 11.1, 3.8 Hz, 3H), 1.54–1.49 (m, 1H), 1.45–1.36 (m, 4H), 1.25 (m, *J* = 14.1, 12.9, 9.4 Hz, 3H), 1.17–1.07 (m, 4H), 0.86 (d, *J* = 7.0 Hz, 3H), 0.70 (d, *J* = 7.0 Hz, 3H). ^13^C-NMR (CDCl_3_) δ ppm 217.03, 167.11, 166.78, 160.81, 158.32, 138.92, 138.58, 131.89, 128.51, 126.24, 117.47, 74.75, 70.53, 58.56, 58.24, 45.57, 44.84, 44.07, 42.00, 36.82, 36.10, 34.56, 30.52, 26.96, 26.52, 24.95, 20.60, 18.54, 16.87, 14.97, 14.32, 11.59. HRMS (ESI) of C_32_H_41_N_3_O_5_S_2_ [M+H]^+^ calcd, 612.2527; Found, 612.2524.

*14-O-[(3-Methylbenzamide-1,3,4-thiadiazol-5-yl) thioacetyl] mutilin* (**5b**): Compound **5a** was prepared according to the general procedure from **4** and *m*-methylbenzoic acid. The crude product was purified over silica gel column chromatography to give **5b** (0.83 g, yield 68%), mp 107–110 °C. IR (KBr): υ_max_ cm^−1^ 3448, 2931, 1732, 1671, 1533, 1458, 1303, 1152, 1116, 1017, 735, 674 cm^−1^. ^1^H-NMR (CDCl_3_) δ ppm 8.03–7.90 (m, 2H), 7.44 (d, *J* = 6.0, 2H), 6.38 (q, *J* = 17.4, 11.0, 1H), 5.74 (d, *J* = 8.4, 1H), 5.30–5.24 (m, 1H), 5.12 (d, *J* = 17.4, 1H), 4.04–3.89 (m, 2H), 3.32 (d, *J* = 6.4, 1H), 2.45 (s, 3H), 2.33–2.17 (m, 3H), 2.01 (t, *J* = 16.0, 8.2, 2H), 1.74 (q, *J* = 14.4, 2.5, 1H), 1.63 (t, *J* = 13.0, 7.2, 2H), 1.51–1.30 (m, 6H), 1.27–1.22 (m, 1H), 1.15–1.05 (m, 4H), 0.85 (d, *J* = 7.0, 3H), 0.68 (d, *J* = 7.0, 3H). ^13^C-NMR (CDCl_3_) δ ppm 217.33, 167.05, 165.88, 161.89, 158.89, 139.25, 134.68, 131.30, 129.58, 129.23, 126.08, 117.78, 75.06, 70.90, 58.55, 45.88, 45.11, 44.37, 42.30, 37.13, 36.52, 34.88, 30.84, 27.26, 26.80, 25.28, 21.81, 17.17, 15.26, 11.92. HRMS (ESI) of C_32_H_41_N_3_O_5_S_2_ [M+H]^+^ calcd, 612.2515; Found, 612.1506.

*14-O-[(4-Methylbenzamide-1,3,4-thiadiazol-5-yl) thioacetyl] mutilin* (**5c**): Compound **5c** was prepared according to the general procedure from **4** and *p*-methylbenzoic acid. The crude product was purified over silica gel column chromatography to give **5c** (0.67 g, yield 55%), mp 82–85 °C. IR (KBr): υ_max_ cm^−1^ 3447, 2931, 1733, 1669, 1612, 1540, 1457, 1375, 1299, 1187, 1116, 1019, 836, 744 cm^−1^. ^1^H-NMR (CDCl_3_) δ ppm 8.10 (d, *J* = 8.2, 2H), 7.34 (d, *J* = 8.1, 2H), 6.37 (q, *J* = 17.4, 11.0, 1H), 5.74 (d, *J* = 8.4, 1H), 5.25 (d, *J* = 11.1, 1H), 5.11 (d, *J* = 17.4, 1H), 4.06–3.90 (m, 2H), 3.31 (s, 1H), 3.20 (s, 1H), 2.45 (s, 3H), 2.30–2.14 (m, 3H), 2.04 (d, *J* = 7.0, 1H), 2.02–1.94 (m, 1H), 1.73 (q, *J* = 14.4, 2.1, 1H), 1.61 (d, *J* = 10.2, 2H), 1.51–1.33 (m, 6H), 1.25 (d, *J* = 15.8, 1H), 1.18 (s, 4H), 1.09 (d, *J* = 6.0, 4H), 0.85 (d, *J* = 6.9, 3H), 0.69 (d, *J* = 7.0, 3H). ^13^C-NMR (CDCl_3_) δ ppm 217.02, 166.73, 165.36, 161.57, 158.45, 144.36, 138.89, 129.69, 128.78, 128.28, 127.52, 117.41, 74.72, 70.55, 58.21, 45.55, 44.04, 41.97, 36.81, 36.10, 34.55, 30.51, 27.02, 26.49, 24.95, 21.85, 21.61, 16.83, 14.93, 11.58. HRMS (ESI) of C_32_H_41_N_3_O_5_S_2_ [M+H]^+^ calcd, 612.2524; Found, 612.2517.

#### 3.2.2. General Procedure for the Synthesis of Compounds **6a**–**d**

Amino acid derivative (5 mmol) was dissolved in a mixture of tetrahydrofuran (50 mL) and water (20 mL). A 1 N NaOH (6 mL) was added followed by the addition of 1.1 g di-*tert*-butyl dicarbonate (5 mmol) dropwise at room temperature. After stirring for 4 h, the tetrahydrofuran was evaporated under vacuum from the reaction mixture. To the residue was added ethyl acetate (50 mL) and 5% citric acid (30 mL). The organic layer was separated, washed with water (20 mL × 3), dried with anhydrous MgSO_4_ and rotary evaporated to dryness. The crude residue was used the next reaction without purification. A mixture of the above N-Boc protected amino acids (2.2 mmol), compound **4** (0.98 g, 2 mmol), 1-ethyl-3-(3-dimethylaminopropyl) carbodiimide hydrochloride (0.42 g, 2.2 mmol), 1-hydroxybenzotriazole (0.30 g, 2.2 mmol), triethylamine (0.30 g, 3.0 mmol) and dichloromethane (50 mL) was stirred at room temperature for 36 h. The mixture was washed with saturated aqueous NaHCO_3_ and water then, evaporated under vacuum and the residue was treated with a mixture of trifluoroacetic acid (TFA, 10 mL) and dichloromethane (10 mL) at room temperature for 30 min. The reaction mixture was quenched with 25% aqueous NaHCO_3_ (30 mL) and washed with water, dried with anhydrous Na_2_SO_4_ overnight and rotary evaporated to dryness. The crude residue thus obtained was purified by silica gel column chromatography (petroleum ether-ethyl acetate 1:2 v/v) to afford the desired compounds.

*14-O-[(3-Methyl-2-(l)-amino-butyrylamide-1,3,4-thioacetyl-5-yl)thioacetyl] mutilin* (**6a**): Compound **6a** was prepared according to the general procedure from **4** and l-valine. The crude product was purified by silica gel column chromatography to give **6a** (0.87 g, yield 72%), mp 91–93 °C. IR (KBr): υ_max_ cm^−1^ 3422, 2932, 1733, 1521, 1458, 1285, 1152, 1117, 1053, 1018, 981, 916, 698. ^1^H-NMR (CDCl_3_) δ ppm 6.41 (dd, *J* = 17.4, 11.0, 1H), 5.74 (d, *J* = 8.5, 1H), 5.30 (dd, *J* = 11.0, 1.3, 1H), 5.16 (dd, *J* = 17.4, 1.4, 1H), 4.11 (q, *J* = 7.1, 1H), 4.00 (s, 1H), 3.54 (d, *J* = 4.0, 1H), 3.34 (d, *J* = 6.4, 1H), 2.40–2.16 (m, 4H), 2.11–1.98 (m, 4H), 1.73 (d, *J* = 2.6, 1H), 1.69–1.56 (m, 3H), 1.55–1.29 (m, 8H), 1.24 (t, *J* = 7.1, 2H), 1.18–1.10 (m, 4H), 1.04 (d, *J* = 7.0, 4H), 0.86 (dd, *J* = 6.9, 1.6, 5H), 0.71 (d, *J* = 7.0, 3H). ^13^C-NMR (CDCl_3_) δ ppm 217.02, 173.25, 166.81, 159.10, 138.94, 117.43, 74.74, 70.58, 60.51, 60.03, 58.24, 45.56, 44.79, 44.10, 41.99, 36.83, 36.22, 34.57, 31.33, 30.53, 26.96, 26.54, 24.95, 19.62, 16.88, 16.39, 14.93, 14.32, 11.58. HRMS (ESI) of C_30_H_46_N_4_O_5_S_2_ [M+H]^+^ calcd, 593.2831; Found, 593.2827.

*14-O-[(3-Methyl-2-(d)-aminobutyrylamide-1,3,4-thioacetyl-5-yl)thioacetyl] mutilin* (**6b**). Compound **6b** was prepared according to the general procedure from **4** and d-valine. The crude product was purified by silica gel column chromatography to give **6b** (0.80 g, yield 66%), mp 82–85 °C. IR (KBr): υ_max_ cm^−1^ 3419, 2935, 1731, 1541, 1456, 1398, 1296, 1153, 1118, 1058, 1018, 980, 915. ^1^H-NMR (DMSO) δ ppm 6.49–6.37 (m, 1H), 5.77 (d, *J* = 8.4 Hz, 1H), 5.32 (dd, *J* = 11.0, 5.9 Hz, 1H), 5.18 (dd, *J* = 17.4, 5.0 Hz, 1H), 4.18–4.08 (m, 1H), 4.02 (d, *J* = 2.7 Hz, 1H), 3.59 (dd, *J* = 42.1, 4.2 Hz, 1H), 3.35 (d, *J* = 6.3 Hz, 1H), 2.37–2.14 (m, 4H), 2.07 (d, *J* = 16.4 Hz, 3H), 1.93 (s, 1H), 1.82–1.73 (m, 1H), 1.70–1.63 (m, 3H), 1.54 (d, *J* = 11.6 Hz, 1H), 1.50–1.33 (m, 6H), 1.27 (t, *J* = 7.1 Hz, 1H), 1.21–1.13 (m, 5H), 1.04 (dd, *J* = 18.6, 6.9 Hz, 4H), 0.88 (dd, *J* = 6.9, 2.2 Hz, 4H), 0.76–0.67 (m, 3H). ^13^C-NMR (DMSO) δ ppm 216.79, 175.22, 168.65, 166.36, 158.38, 138.44, 116.94, 74.23, 70.08, 61.50, 60.10, 57.74, 45.48, 45.09, 44.21, 43.60, 41.50, 36.34, 35.67, 34.12, 30.03, 26.47, 26.10, 24.46, 20.70, 16.43, 14.46, 13.87, 11.18. HRMS (ESI) of C_30_H_46_N_4_O_5_S_2_ [M+H]^+^ calcd, calcd, 593.2833; Found, 593.2828.

*14-O-[(2-d(−)Phenylglycinamide*-*1,3,4-thioacetyl-5-yl)thioacetyl] mutilin* (**6c**): Compound **6c** was prepared according to the general procedure from **4** and d-phenylglycine. The crude product was purified by silica gel column chromatography to give **6c** (0.72 g, yield 58%), mp 89–91 °C. IR (KBr): υ_max_ cm^−1^ 3448, 2927, 1730, 1522, 1457, 1285, 1153, 1116, 1017, 937, 916, 698. ^1^H-NMR (CDCl_3_) δ ppm 7.43 (d, *J* = 7.2 Hz, 2H), 7.36–7.27 (m, 3H), 6.42 (dd, *J* = 17.3, 11.0 Hz, 1H), 5.72 (d, *J* = 8.4 Hz, 1H), 5.20 (dd, *J* = 26.9, 14.2 Hz, 2H), 4.58 (s, 1H), 3.35 (d, *J* = 6.4 Hz, 1H), 3.27–3.04 (m, 5H), 2.37–2.28 (m, 1H), 2.22 (dd, *J* = 17.2, 9.5 Hz, 2H), 2.11–2.01 (m, 4H), 1.76 (t, *J* = 12.9 Hz, 1H), 1.64 (dd, *J* = 22.0, 11.3 Hz, 2H), 1.56–1.35 (m, 4H), 1.26 (dd, *J* = 15.6, 10.8 Hz, 2H), 1.19–1.09 (m, 7H), 0.89 (d, *J* = 7.0 Hz, 3H), 0.70 (t, *J* = 8.9 Hz, 3H). ^13^C-NMR (CDCl_3_) δ ppm 217.18, 173.23, 170.02, 139.19, 129.07, 128.30, 127.19, 117.48, 74.81, 69.97, 60.02, 58.39, 47.77, 47.40, 45.67, 45.07, 44.17, 42.02, 36.96, 36.24, 34.67, 31.68, 30.66, 27.09, 26.54, 25.07, 17.11, 15.12, 14.41, 11.76. HRMS (ESI) of C_32_H_42_N_4_O_5_S_2_ [M+H]^+^ calcd, 627.2663; found, 627.2659.

*14-O-[(2-l(−)Phenylglycinamide-1,3,4-thioacetyl-5-yl)thioacetyl] mutilin* (**6d**): Compound **6d** was prepared according to the general procedure from **4** and l-phenylglycine. The crude product was purified over silica gel column chromatography to give **6d** (0.79 g, yield 63%), mp 86–89 °C. IR (KBr): υ_max_ cm^−1^ 3447, 2927, 1731, 1522, 1457, 1284, 1153, 1117, 1018, 980, 938, 916, 699. ^1^H-NMR (CDCl_3_) δ ppm 7.36 (m, *J* = 13.5, 9.7, 4.9 Hz, 5H), 6.39 (dd, *J* = 17.4, 11.0 Hz, 1H), 5.73 (d, *J* = 8.5 Hz, 1H), 5.27 (d, *J* = 10.9 Hz, 1H), 5.13 (dd, *J* = 17.4, 1.5 Hz, 1H), 4.85 (s, 1H), 4.10 (t, *J* = 7.1 Hz, 1H), 3.98 (s, 2H), 3.33 (d, *J* = 6.4 Hz, 1H), 2.31–2.14 (m, 3H), 2.09–1.98 (m, 4H), 1.72 (d, *J* = 2.5 Hz, 1H), 1.67–1.57 (m, 2H), 1.52–1.39 (m, 5H), 1.33 (d, *J* = 13.6 Hz, 1H), 1.25 (t, *J* = 7.1 Hz, 2H), 1.15–1.07 (m, 4H), 0.85 (d, *J* = 7.0 Hz, 3H), 0.70 (dd, *J* = 7.0, 2.1 Hz, 3H). ^13^C-NMR (CDCl_3_) δ ppm 217.43, 172.14, 171.69, 167.20, 139.71, 139.35, 129.74, 129.24, 127.40, 117.84, 77.50, 77.26, 75.14, 71.00, 60.93, 59.96, 58.64, 45.97, 45.18, 44.51, 42.41, 37.24, 36.63, 34.99 (s), 30.94, 27.37, 26.92, 25.37, 21.58, 17.30, 15.36, 14.74, 12.01. HRMS (ESI) of C_32_H_42_N_4_O_5_S_2_ [M+H]^+^ calcd, 627.2669; Found, 627.2661.

### 3.3. Antibacterial Activity

#### 3.3.1. MIC Determination

The MIC studies were performed on MRSA, MRSE, *S. aureus* CVCC 1882, *S. epidermidis* CMCC 26069, *E. coli* CVCC 1570, and *B. cereus* CMCC (B) 63302 using the agar dilution method according to the National Committee for Clinical Laboratory Standards (NCCLS). Compounds were dissolved in 25%–45% DMSO in water to prepare a solution that had a concentration of 1.28 mg/mL. Tiamulin fumarate used as reference drug was directly dissolved in 10 mL distilled water. All the solutions were then diluted two-fold with distilled water to provide 11 dilutions down to the final concentration (0.625 μg/mL). The dilutions (2 mL) of each test compound/drug were incorporated into 18 mL hot Mueller-Hinton agar medium, which resulted in the final concentration of each dilutions decreasing tenfold.

Inoculums of MRSA, MRSE, *S. aureus*, *S. epidermidis*, *E. coli*, and *B. cereus* were prepared from blood slants and adjusted to approximately 10^5^–10^6^ CFU/mL with sterile saline (0.90% NaCl). A 10 μL amount of bacterial suspension was spotted onto Mueller-Hinton agar plates containing serial dilutions of the compounds/drugs. The plates were incubated at 36.5 °C for 24–48 h. The MIC was defined as the minimum concentration of the compound needed to completely inhibit bacterial growth. The same procedure was repeated in triplicate.

#### 3.3.2. Oxford Cup Assay

Oxford cup assays were performed to evaluate the rate of inhibition of bacterial growth. Inoculums were prepared in 0.9% saline using McFarland standard and spread uniformly on Mueller-Hinton agar plates. All the compounds were diluted to 320 and 160 μg/mL and the resulting solutions were added to the Oxford cups which were placed at equal distances above agar surfaces. The zone of inhibition for each concentration was measured after 24 h incubation at 37 °C. The same procedure was repeated in triplicate.

### 3.4. Molecular Modeling Studies

The crystal structure of 50S ribosomal subunit from *Deinococcus radiodurans* in complex with tiamulin (PDB ID: 1XBP) [10] was used for all simulations with Homdock software in the Chil^2^ package [17] combining of a Graph based molecular alignment (GMA) tool and a Monte-Carlo/Simulated Annealing (MC/SA) algorithm based docking (GlamDock) tool. Tiamulin was the template for flexible molecular alignment, and the interaction was optimized by GlamDock according to the ChillScore scoring function based on ChemScore with a smooth, improved potential. All the compounds were prepared with Avogadro software [21], including a 5,000 steps Steepest Descent and 1000 steps Conjugate Gradients geometry optimization based on the MMFF94 force field. The 50S ribosomal subunit was extracted from the crystal structure of 1XBP and transformed to mol2 format. The docking position was set to the binding site of tiamulin. All the compounds were superposed to tiamulin as the template by the GMA, and the placement of compounds were subjected to a 200 steps’ gradient optimization by Glamdock engine. All the other parameters were set to be default.

As a result of calculations, each compound, which has the best RMSD compared to tiamulin conformation, was kept for binding affinity comparison. The binding affinity between compounds and receptor were estimated by ChillScore. Hydrogen bonds and other interactions were detected by PoseView [22] and all the figures were generated by PyMol 1.5.03 [23].

### 3.5. ADMET Prediction

The prediction of ADMET properties is important in the drug selection and promotion process so as to avoid inappropriate compounds before substantial time and money are invested in testing [24,25]. The pharmacokinetic and toxic profiles of the tested compounds were predicted by the ADMET descriptors protocol in ACD/Labs. The parameters with higher prediction accuracy and/or Reliability Index values (RI; a number ranging from 0 to 1 (0: Unreliable prediction, 1: Ideal, fully reliable prediction), is used to estimate reliability of prediction.) containing absorption, Log BB, Log PS, PPB, Vd and LD_50_ as ADMET descriptors, were selected and predicted using ACD/Percepta Platform available online. In order to further understand the above-mentioned properties, pKa and Log *p* also were predicted.

## 4. Conclusions

In summary, we have synthesized seven new pleuromutilin derivatives containing thiadiazole moieties. In evaluation of the *in vivo* antibacterial activity of the synthesized compounds, all the synthesized compounds showed moderate to good inhibitory characteristics. Compound **6d**, bearing a l(−)phenylglycinamide group on the C-14 glycolic acid side chain, was the most active antibacterial agents against MRSA (MIC = 0.5 μg/mL), MRSE (MIC = 4 μg/mL), *S. aureus* (MIC = 0.5 μg/mL), *S. epidermidis* (MIC = 2 μg/mL) and *B. cereus* (MIC = 0.25 μg/mL). Docking studies revealed that the binding free energies (ΔG_b_) were in the range of −10.42 to −15.09 kcal/mol, with an RMSD range of 1.00 to 1.23 Å. Compound **6d** with the most antibacterial activity displayed the highest binding affinity (ΔG_b_ = −15.09). All of the compounds were further predicted their ADMET properties including absorption, Log BB, Log PS, PPB, Vd and LD_50_, as well as pKa and Log *p*. The results indicate that the seven compounds might have moderate to good ADMET properties and the compound **6d** which shows the highest antibacterial activity among them may be a drug-like compound.

## Supplementary Materials

Supplementary materials can be accessed at: http://www.mdpi.com/1420-3049/19/11/19050/s1.
