# Supplementary material for: Synthesis and Biological Evaluation of New Pleuromutilin Derivatives as Antibacterial Agents
Source: Molecules. 2014 Nov 19;19(11):19050–65. doi: 10.3390/molecules191119050 (PMC6271455; doi:10.3390/molecules191119050)

# Supplementary Materials

**Figure S1.** IR spectrum of compound 2.

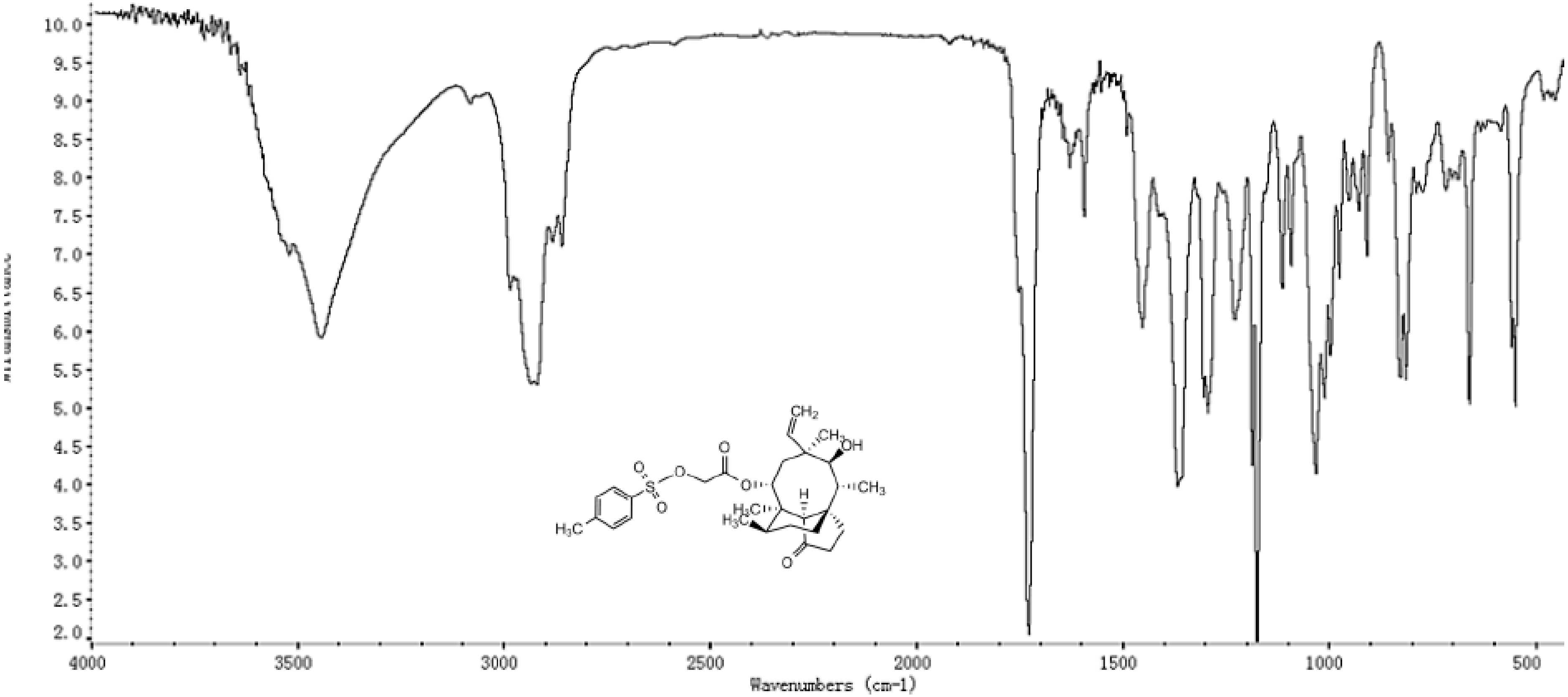

**Figure S2.** $^{1}$H-NMR spectrum of compound 2.

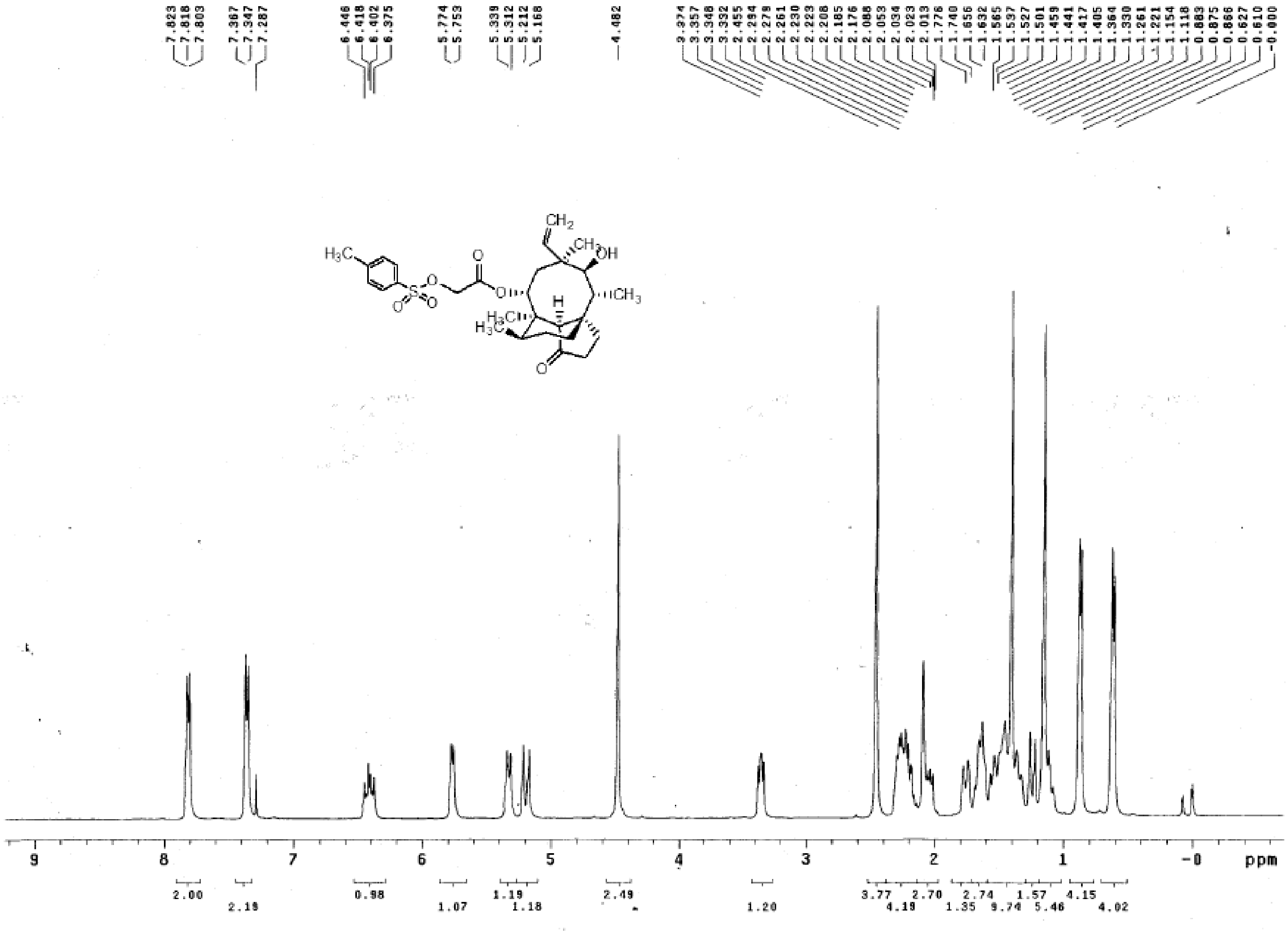

**Figure S3.** $^{13}$C-NMR spectrum of compound 2.

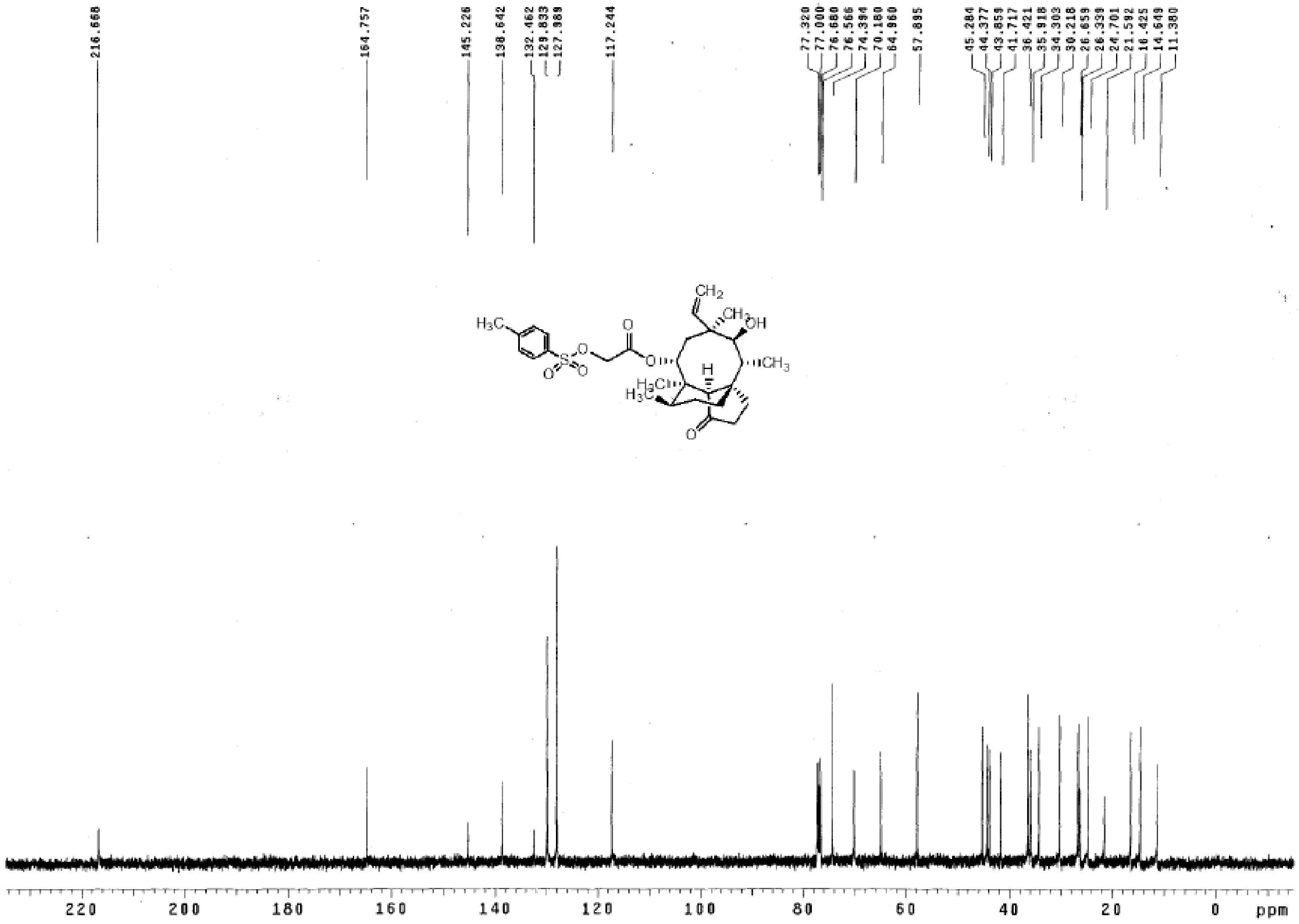

**Figure S4.** IR spectrum of compound 3.

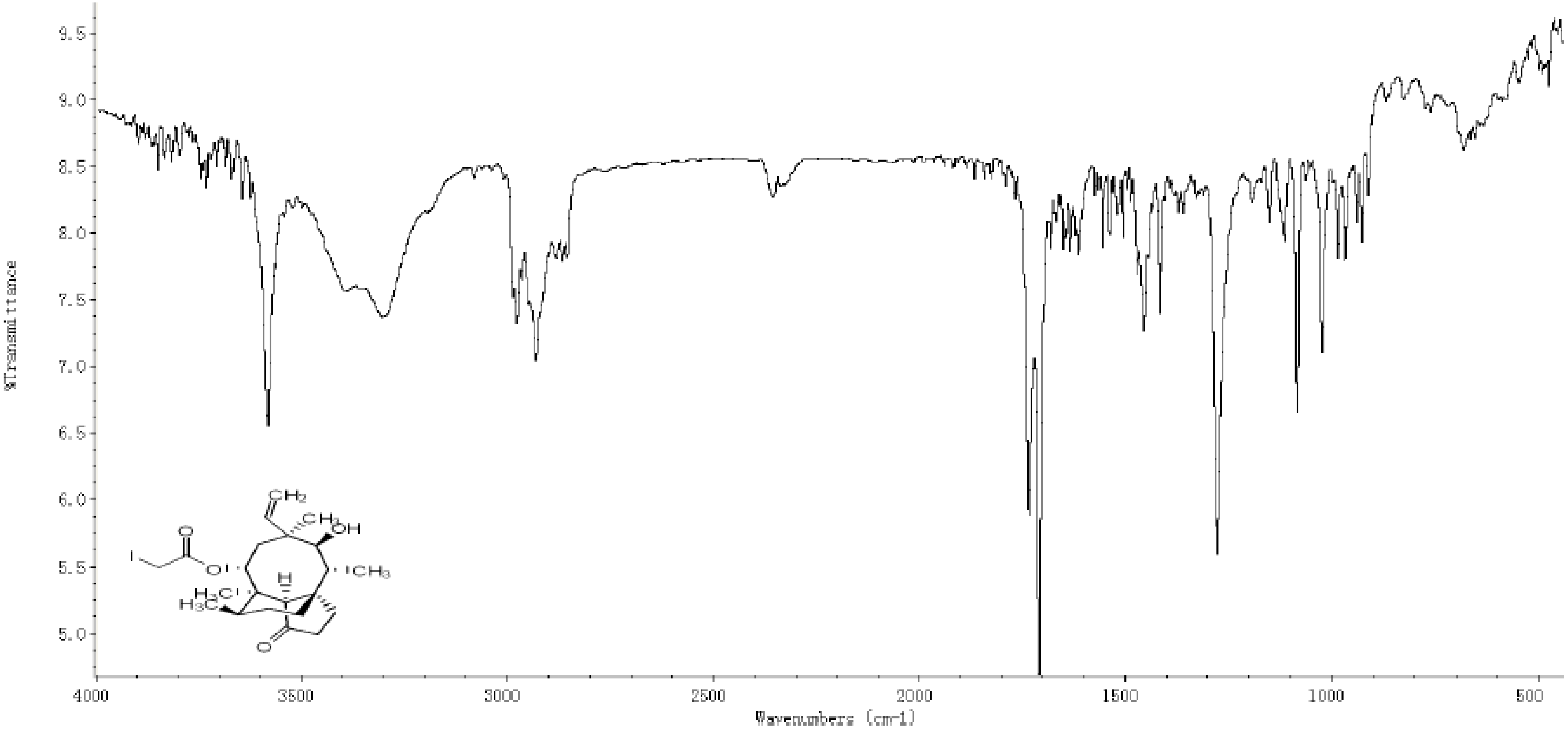

**Figure S5.** $^1$H-NMR spectrum of compound 3.

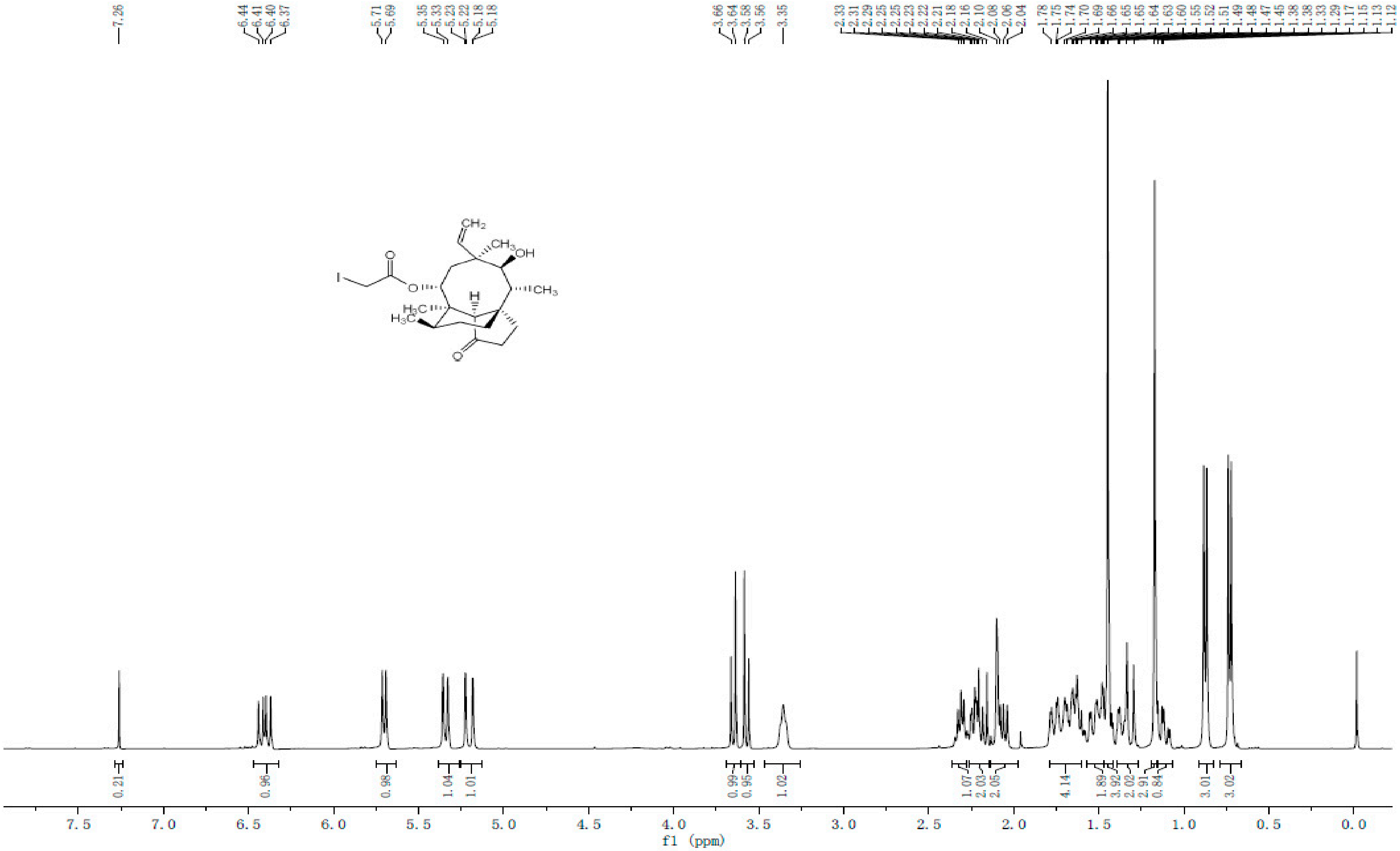

**Figure S6.** $^{13}$C-NMR spectrum of compound 3.

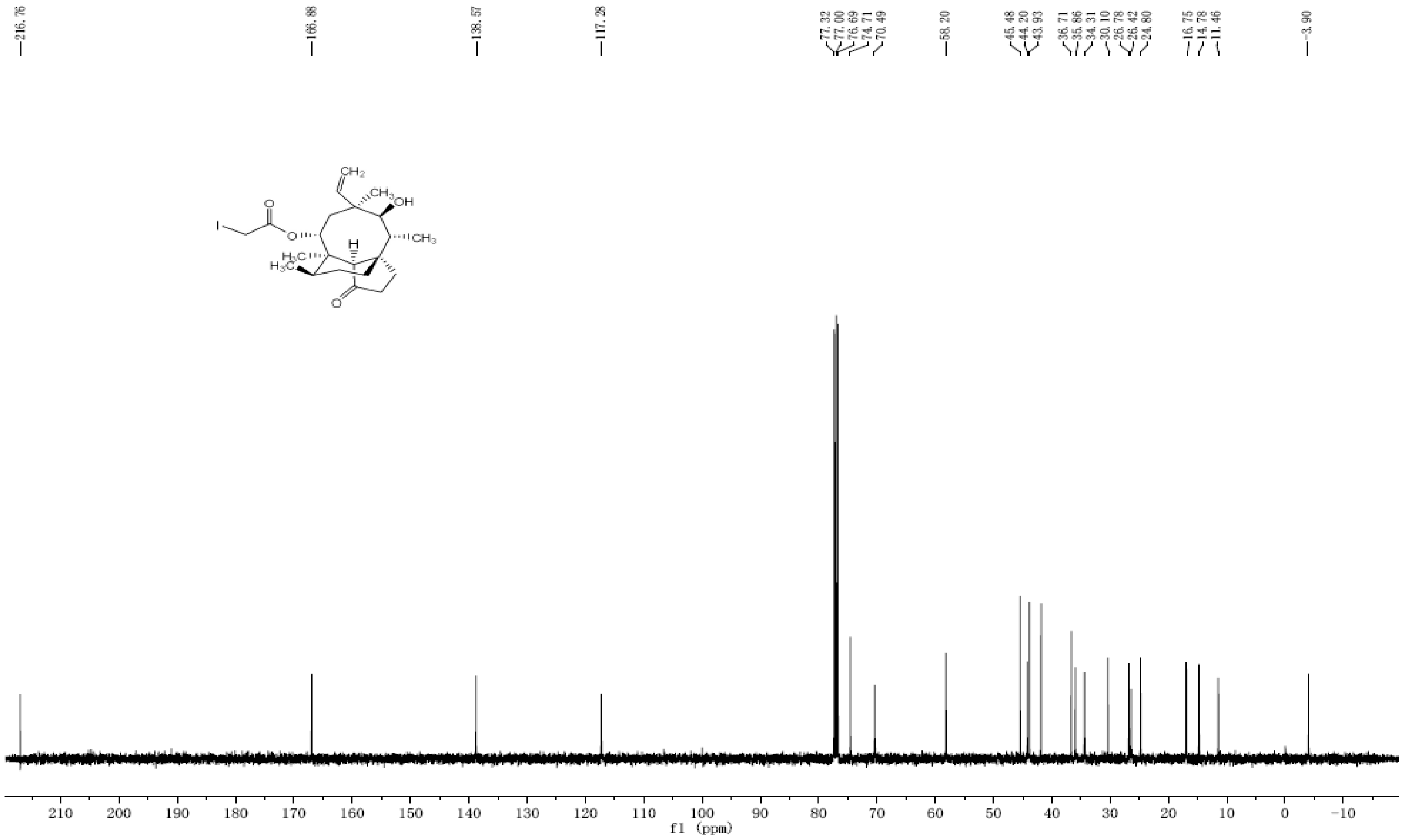

**Figure S7.** IR spectrum of compound 4.

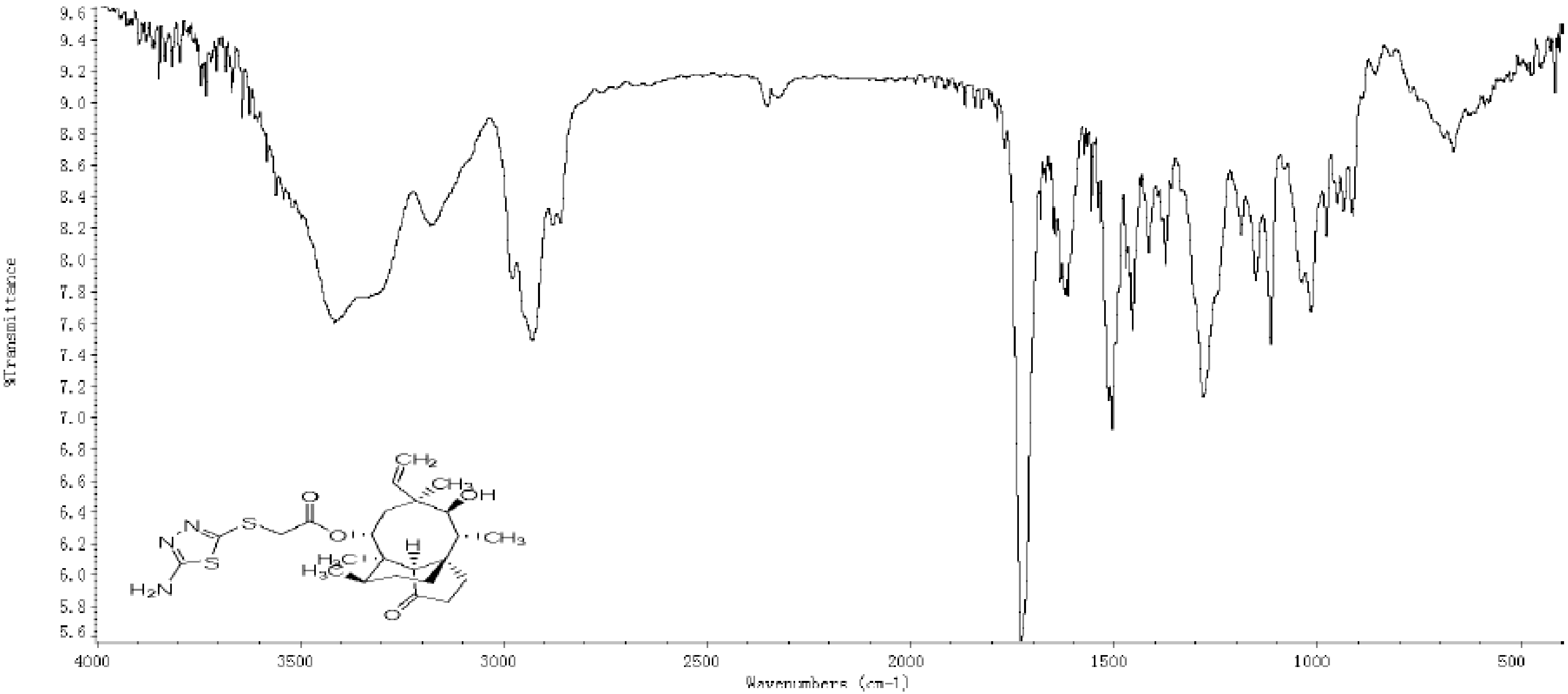

**Figure S8.** $^{1}$H-NMR spectrum of compound 4.

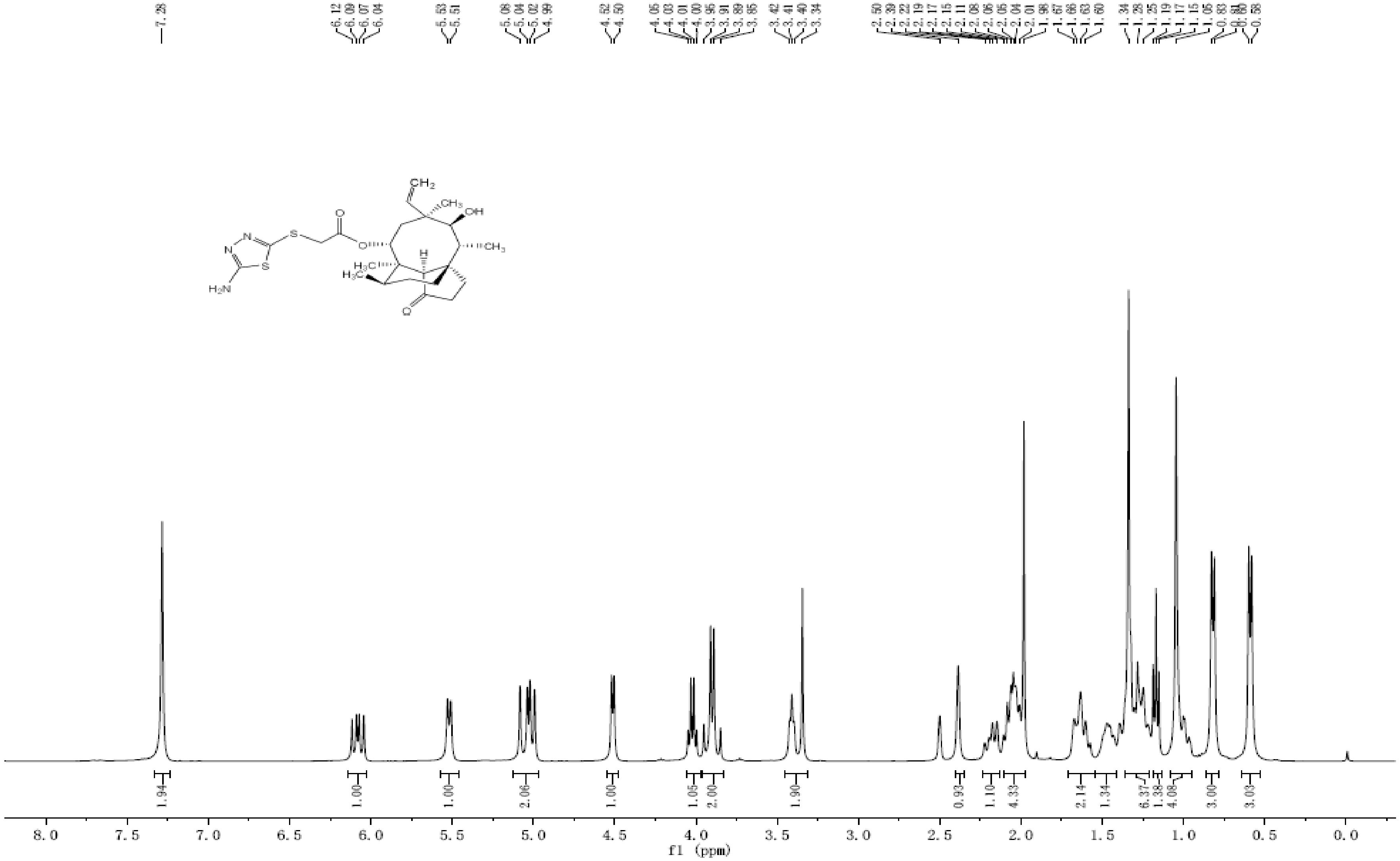

**Figure S9.** $^{13}$C-NMR spectrum of compound 4.

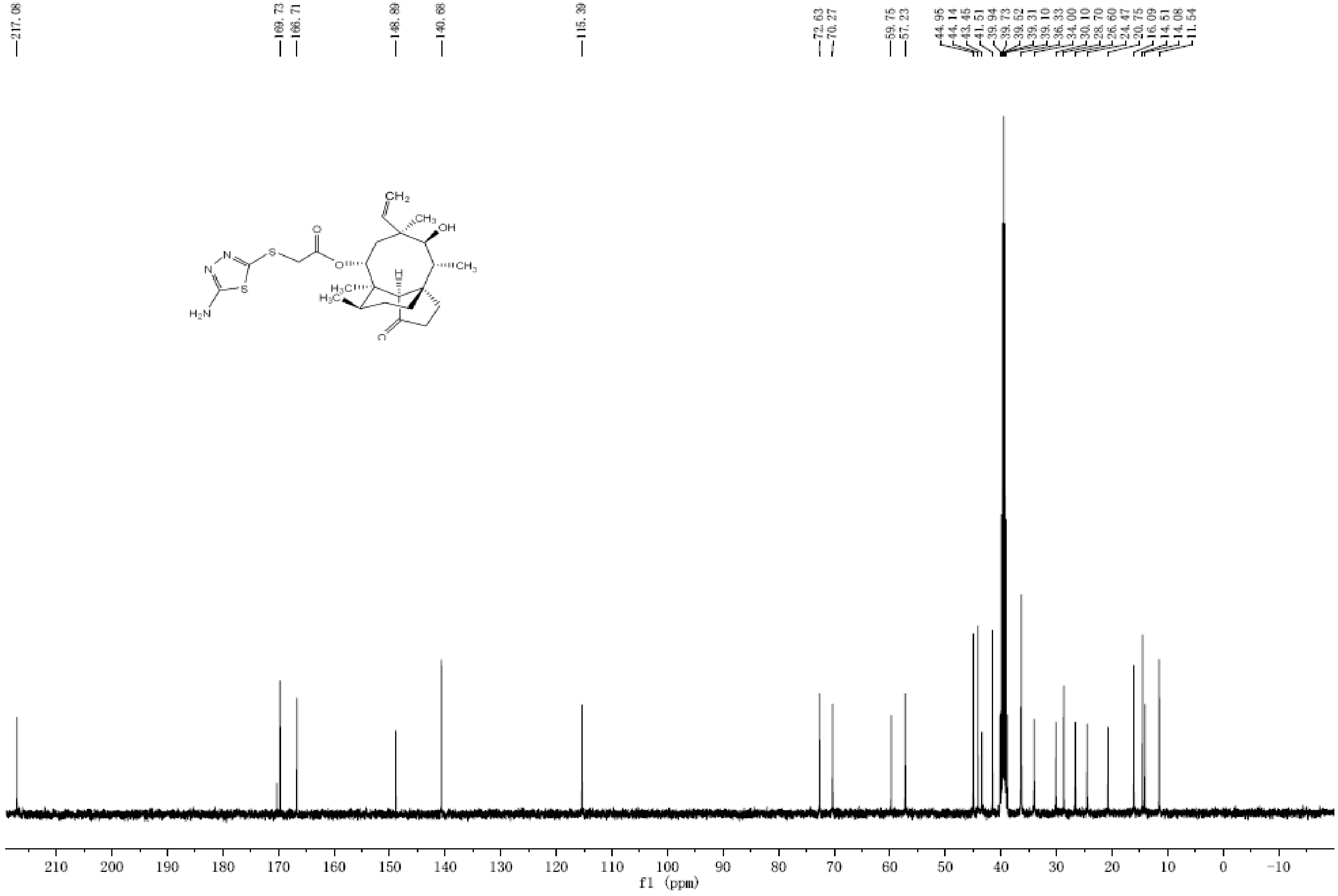

**Figure S10.** IR spectrum of compound 5a.

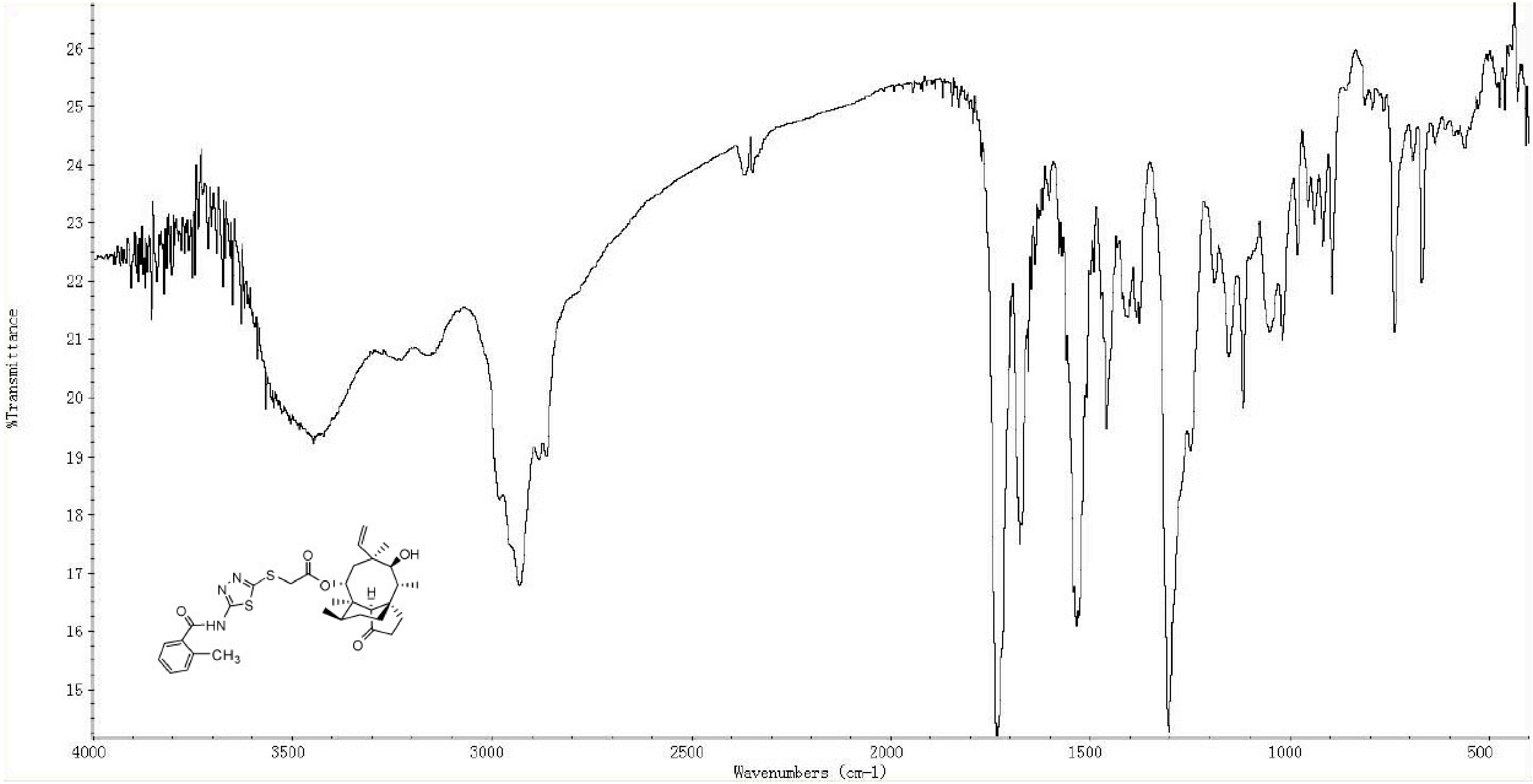

**Figure S11.** $^{1}$H NMR spectrum of compound 5a.

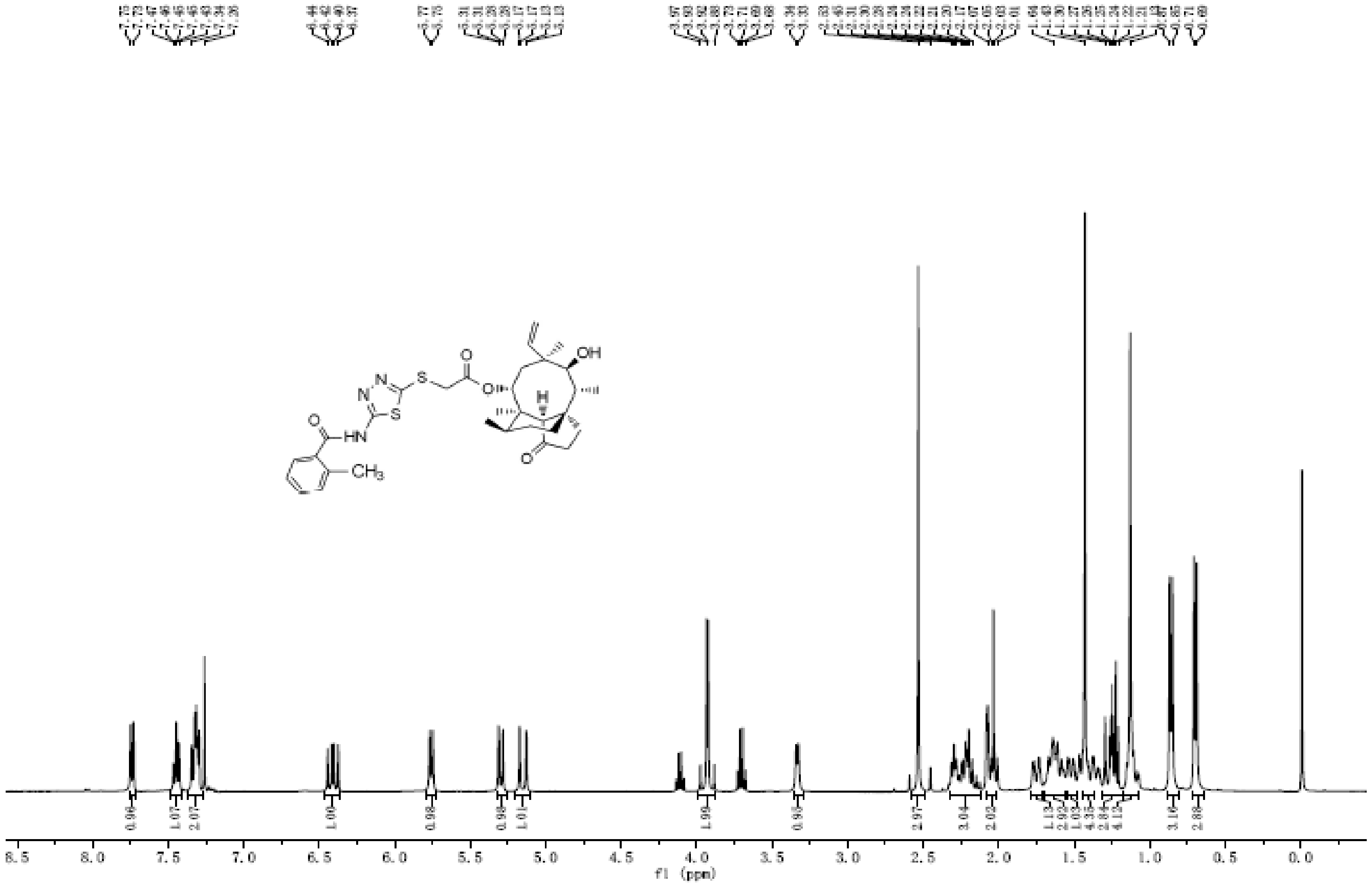

**Figure S12.** $^{13}$C-NMR spectrum of compound 5a.

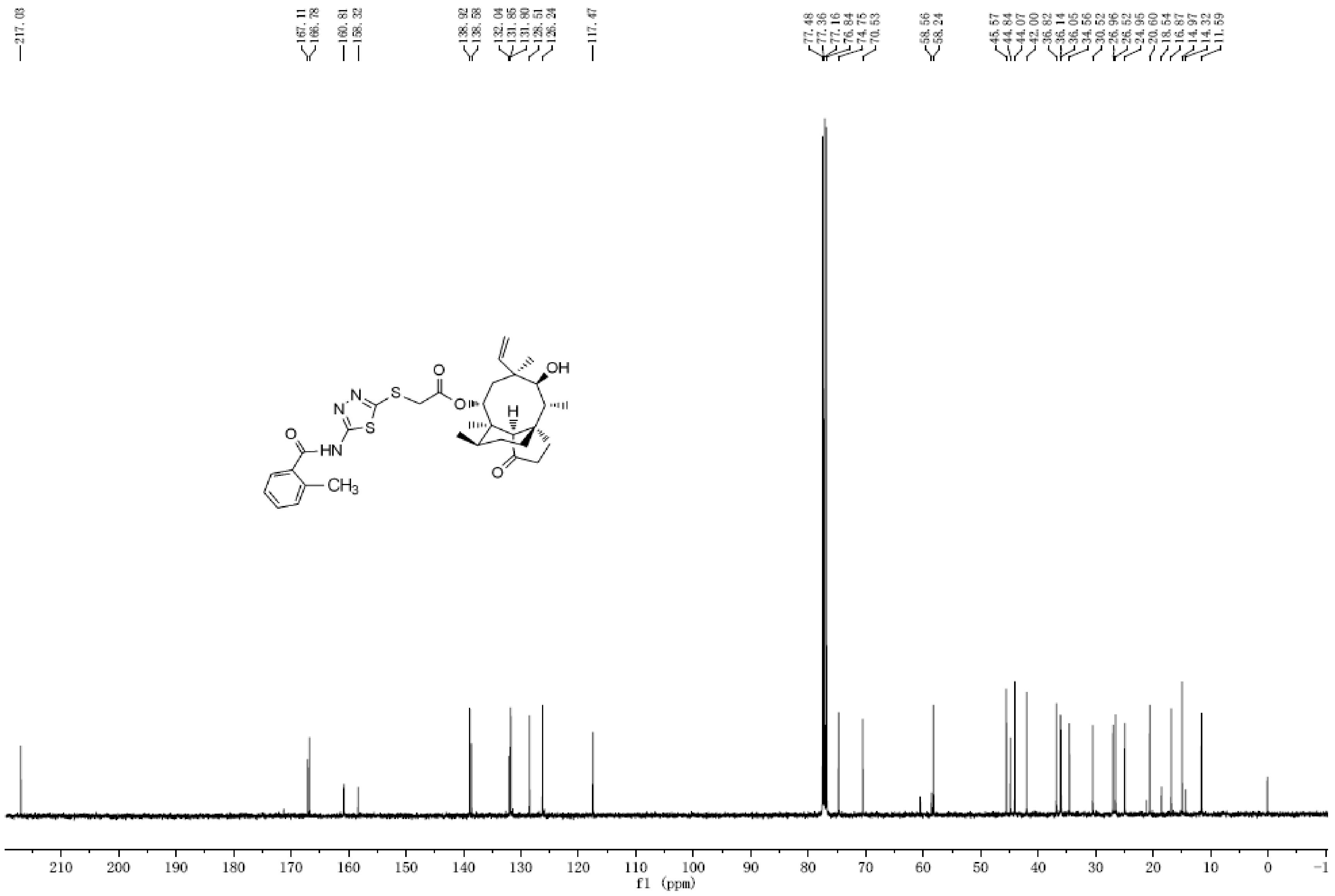

**Figure S13.** IR spectrum of compound 5b.

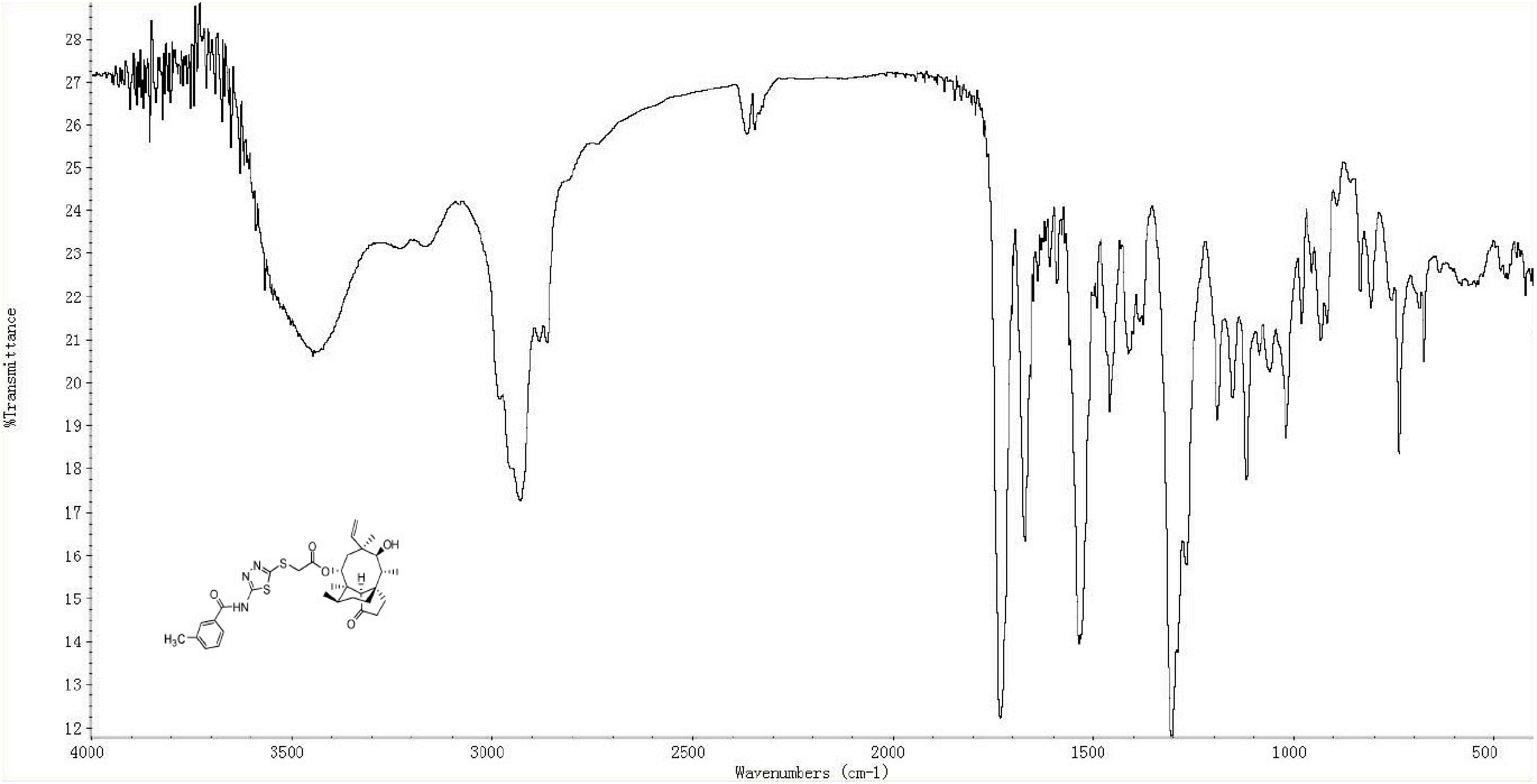

**Figure S14.** $^{1}$H-NMR spectrum of compound 5b.

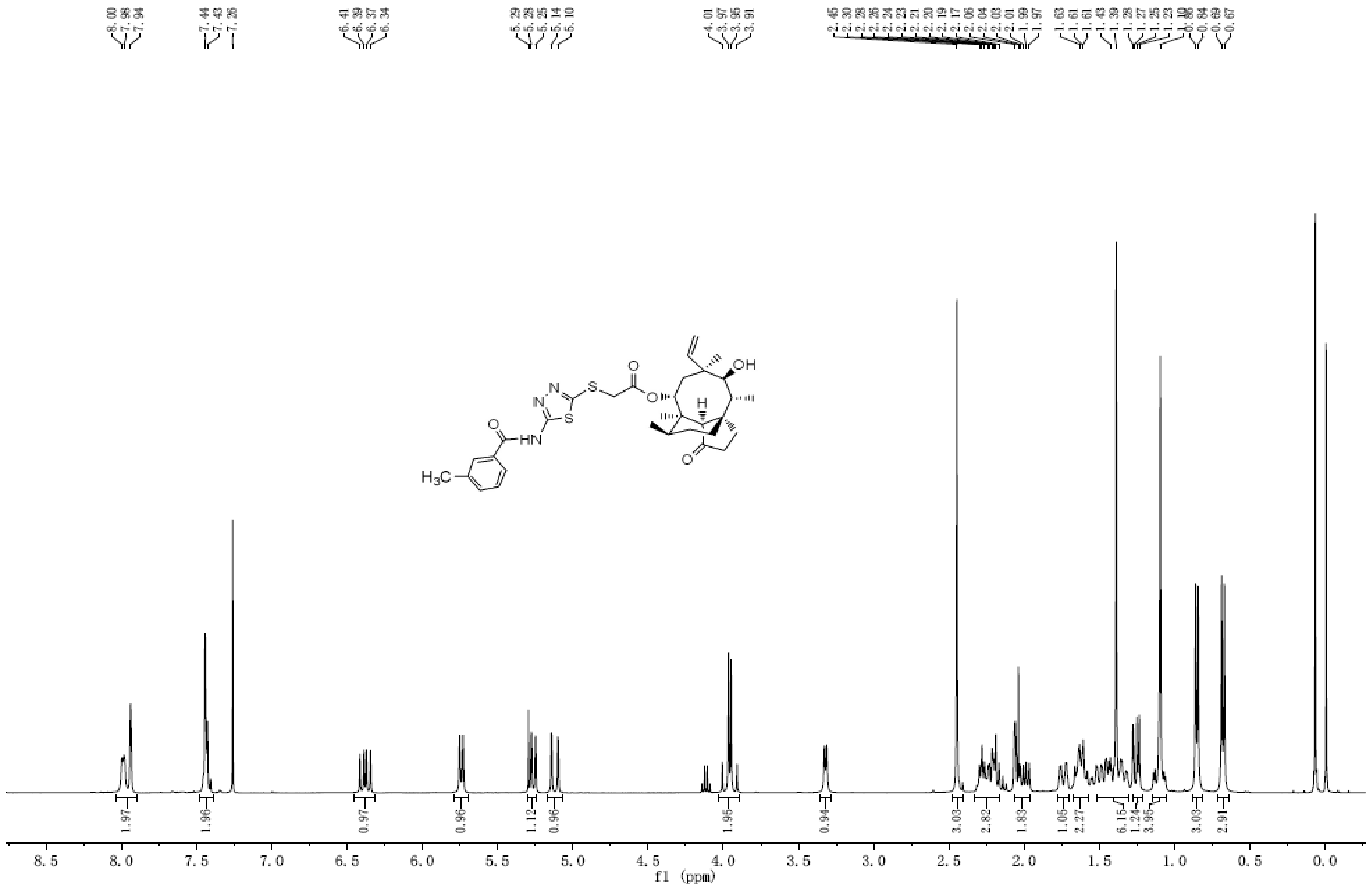

**Figure S15.** $^{13}C$ NMR spectrum of compound 5b.

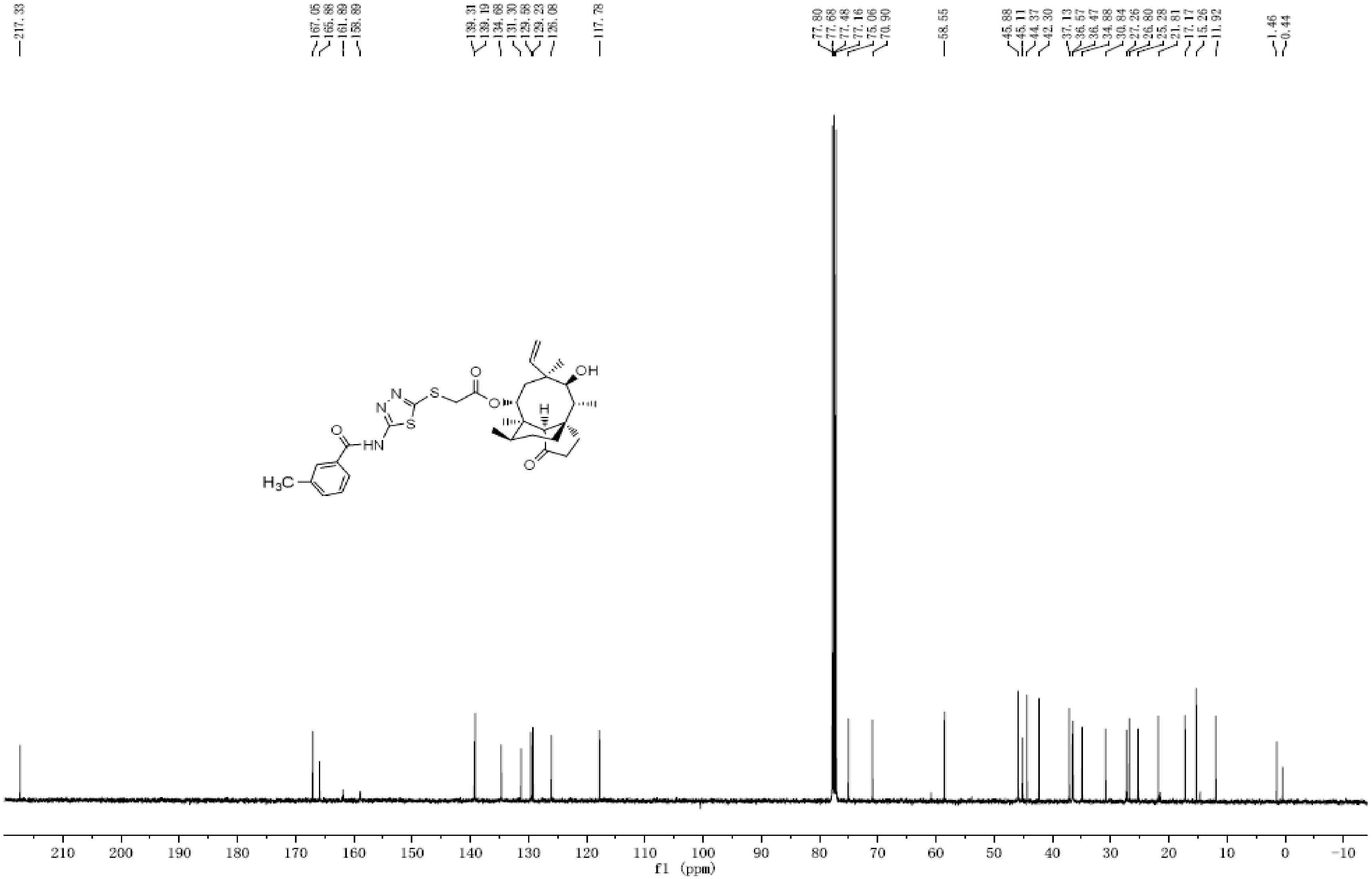

**Figure S16.** IR spectrum of compound 5c.

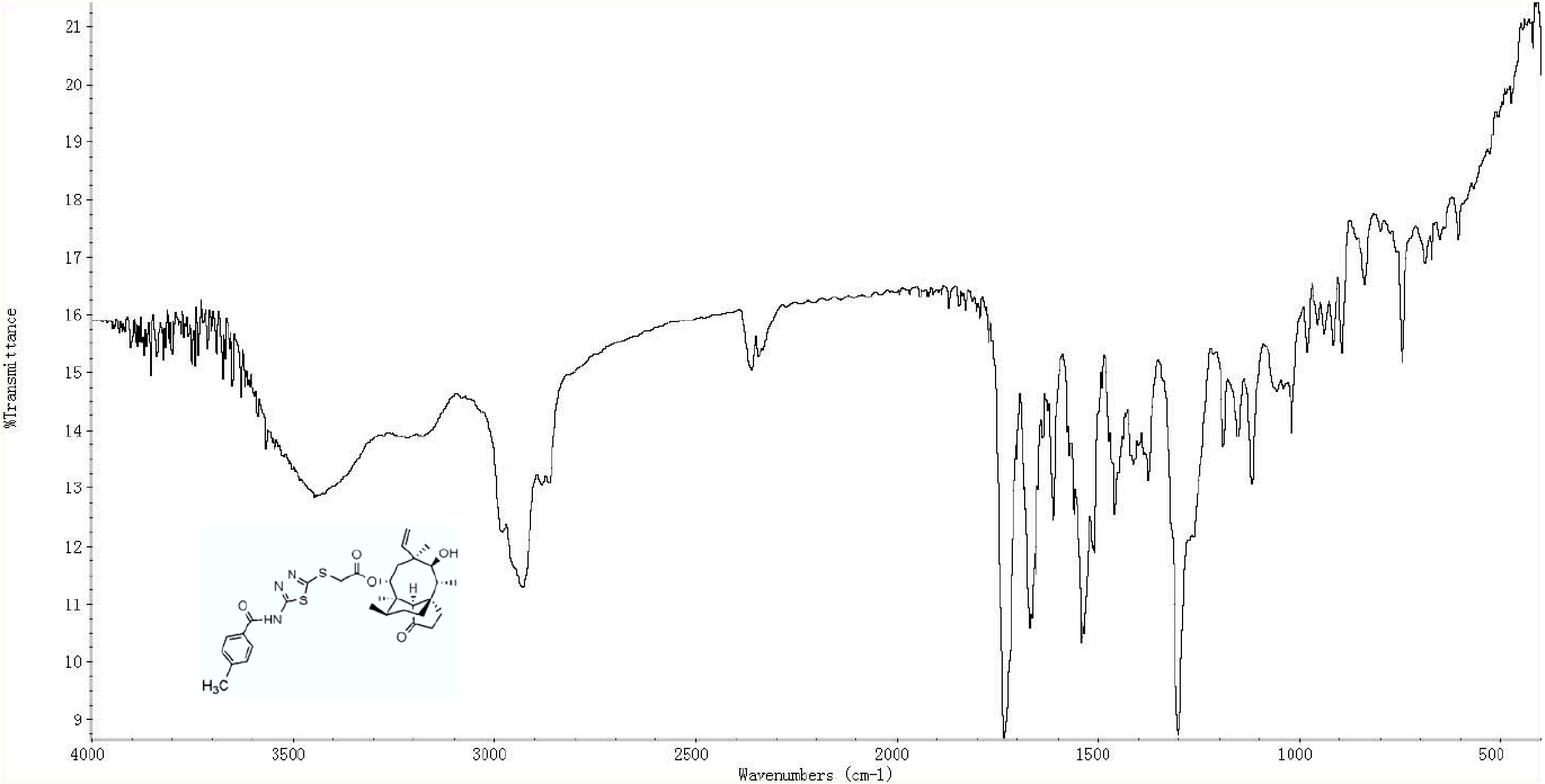

**Figure S17.** $^{1}$H-NMR spectrum of compound 5c.

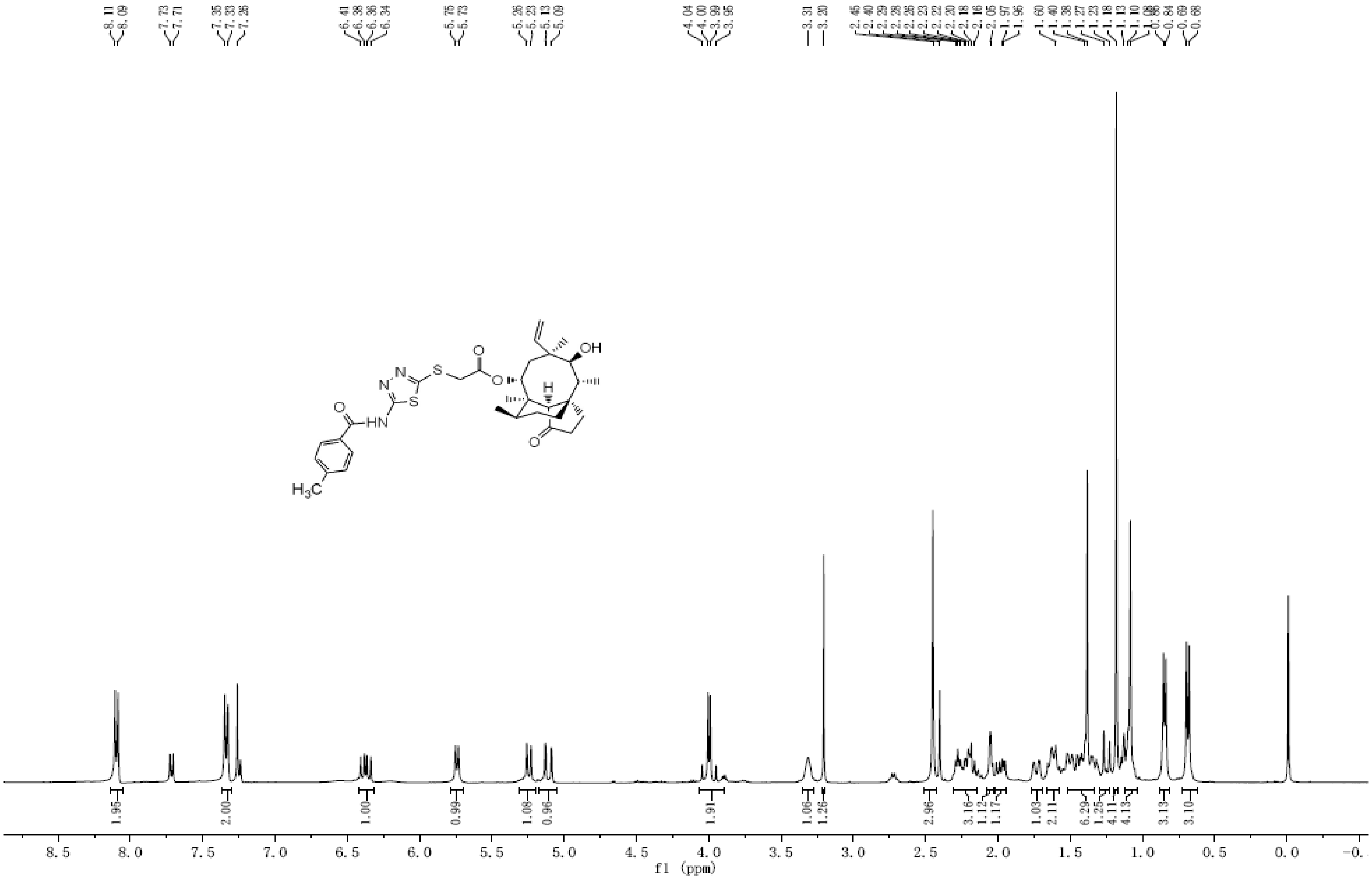

**Figure S18.** $^{13}$C NMR spectrum of compound 5c.

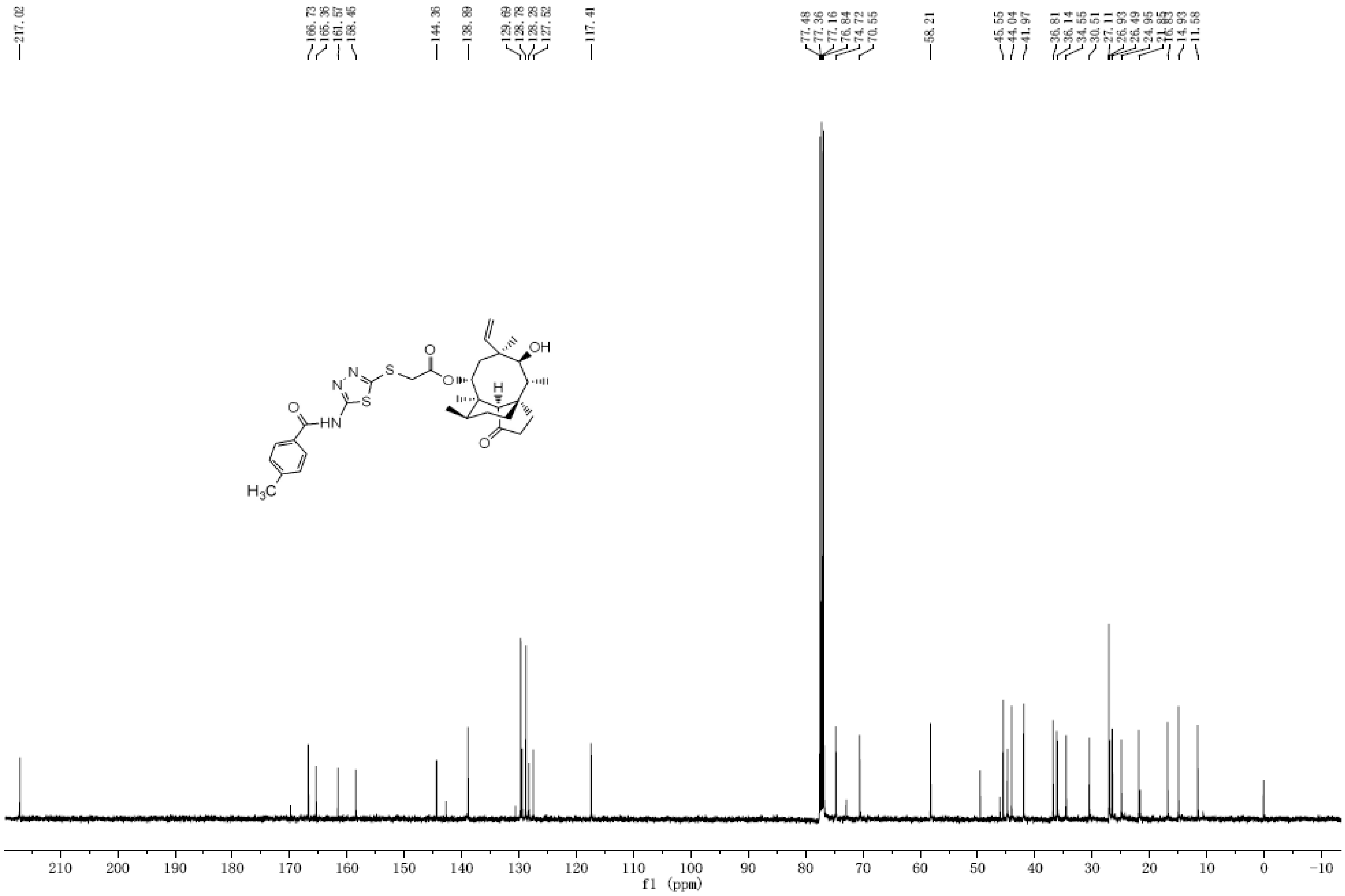

**Figure S19.** IR spectrum of compound 6a.

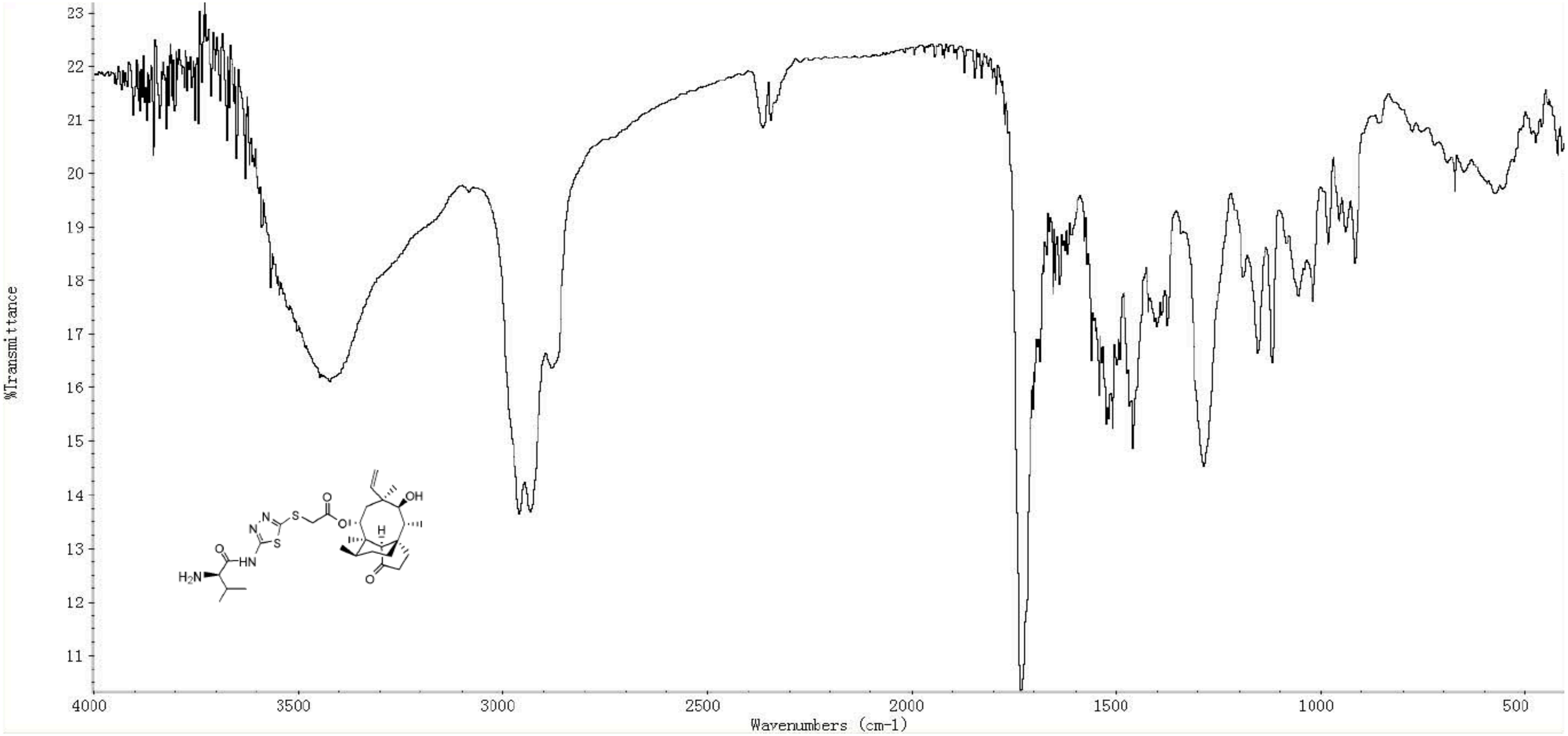

**Figure S20.** $^1$H NMR spectrum of compound 6a.

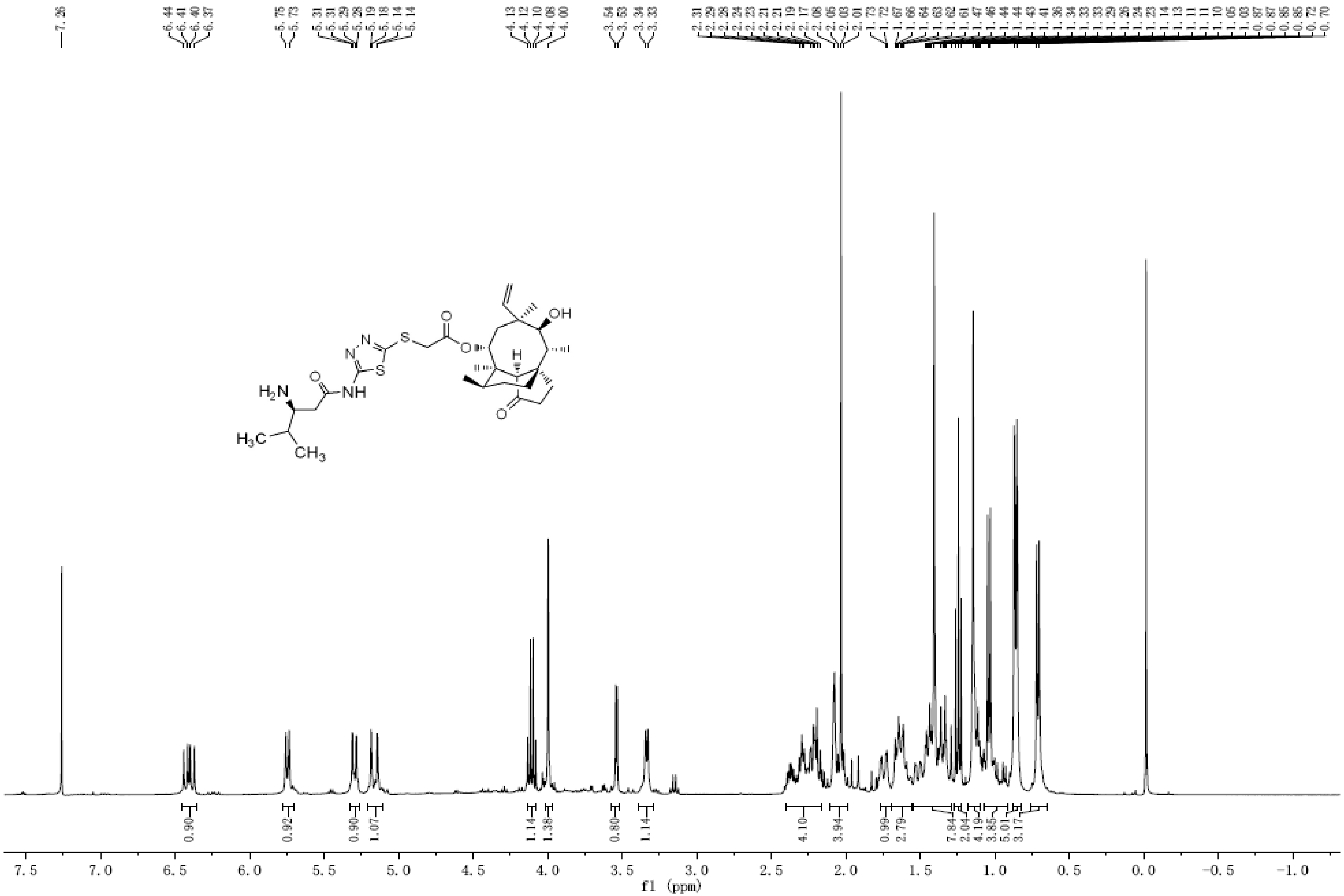

**Figure S21.** $^{13}C$ NMR spectrum of compound 6a.

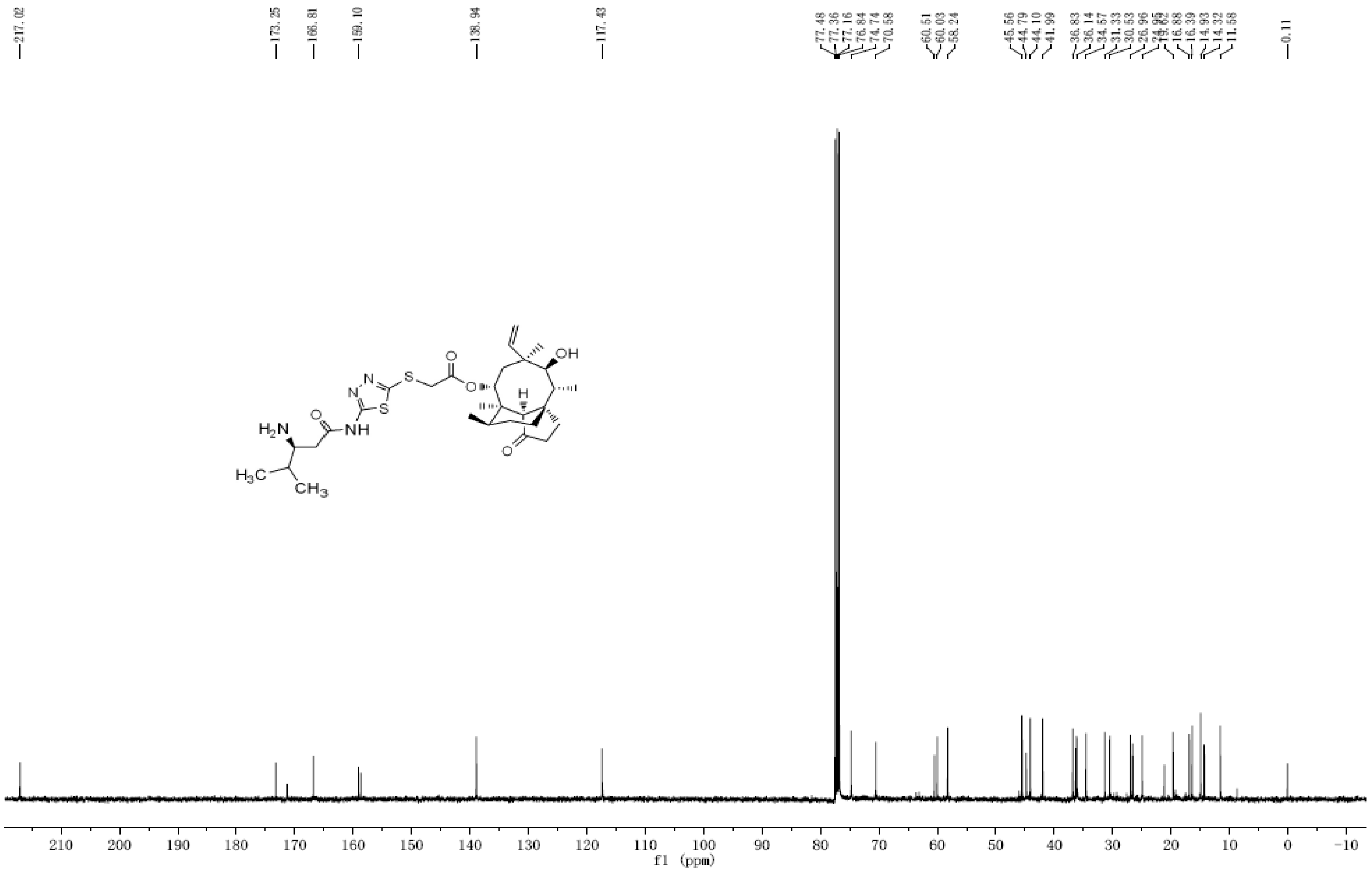

**Figure S22.** IR spectrum of compound 6b.

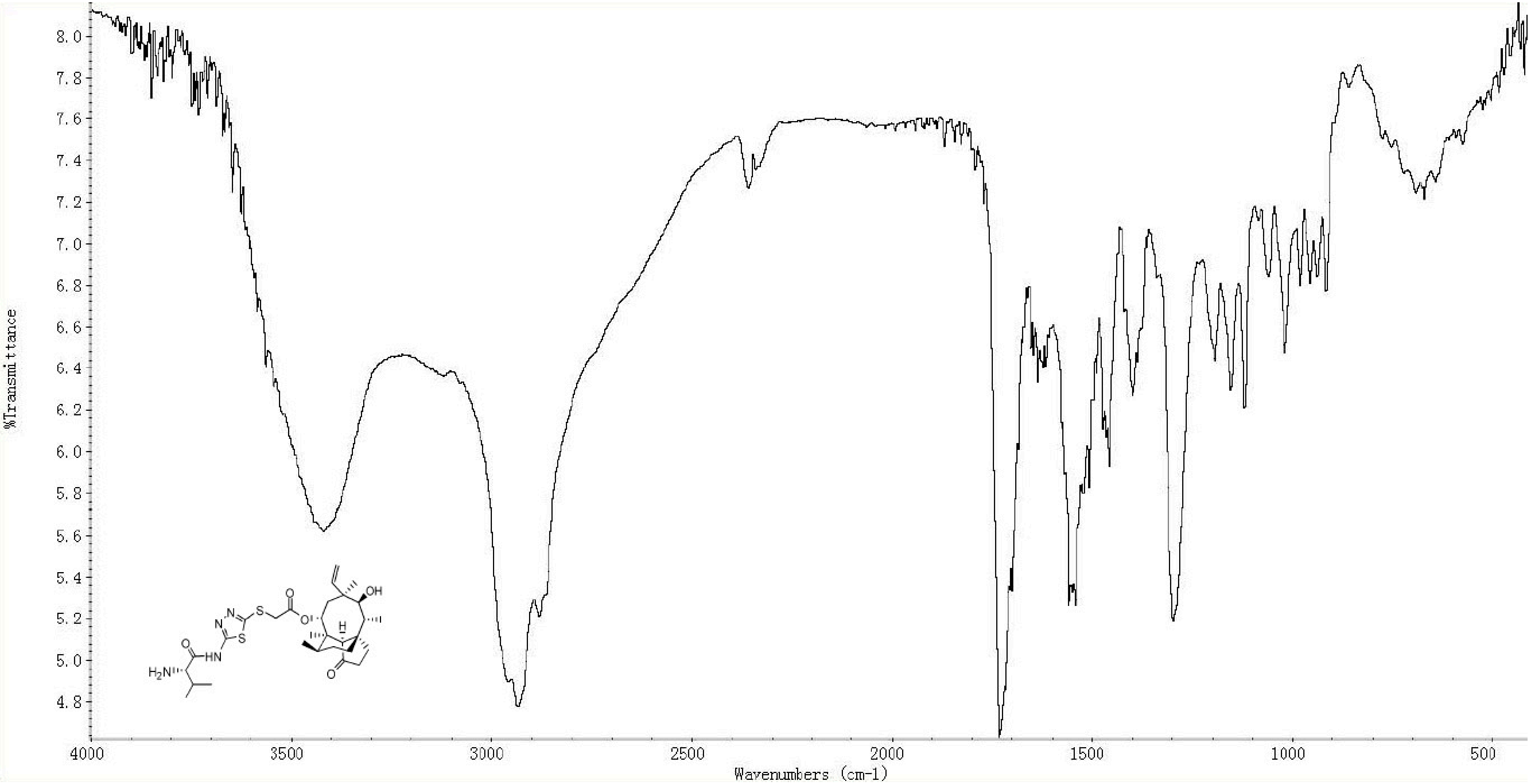

**Figure S23.** $^{1}$H NMR spectrum of compound 6b.

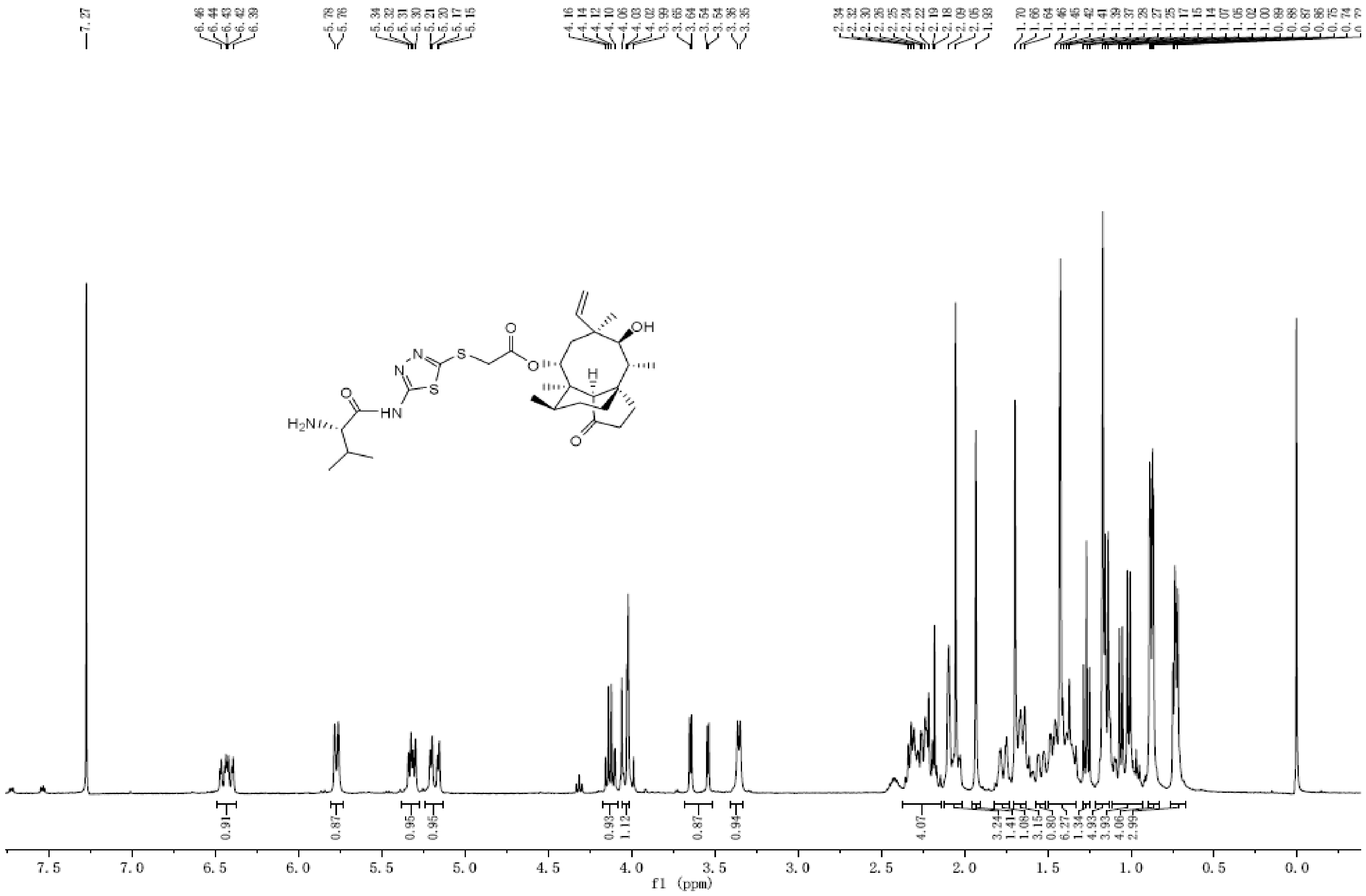

**Figure S24.** $^{13}C$ NMR spectrum of compound 6b.

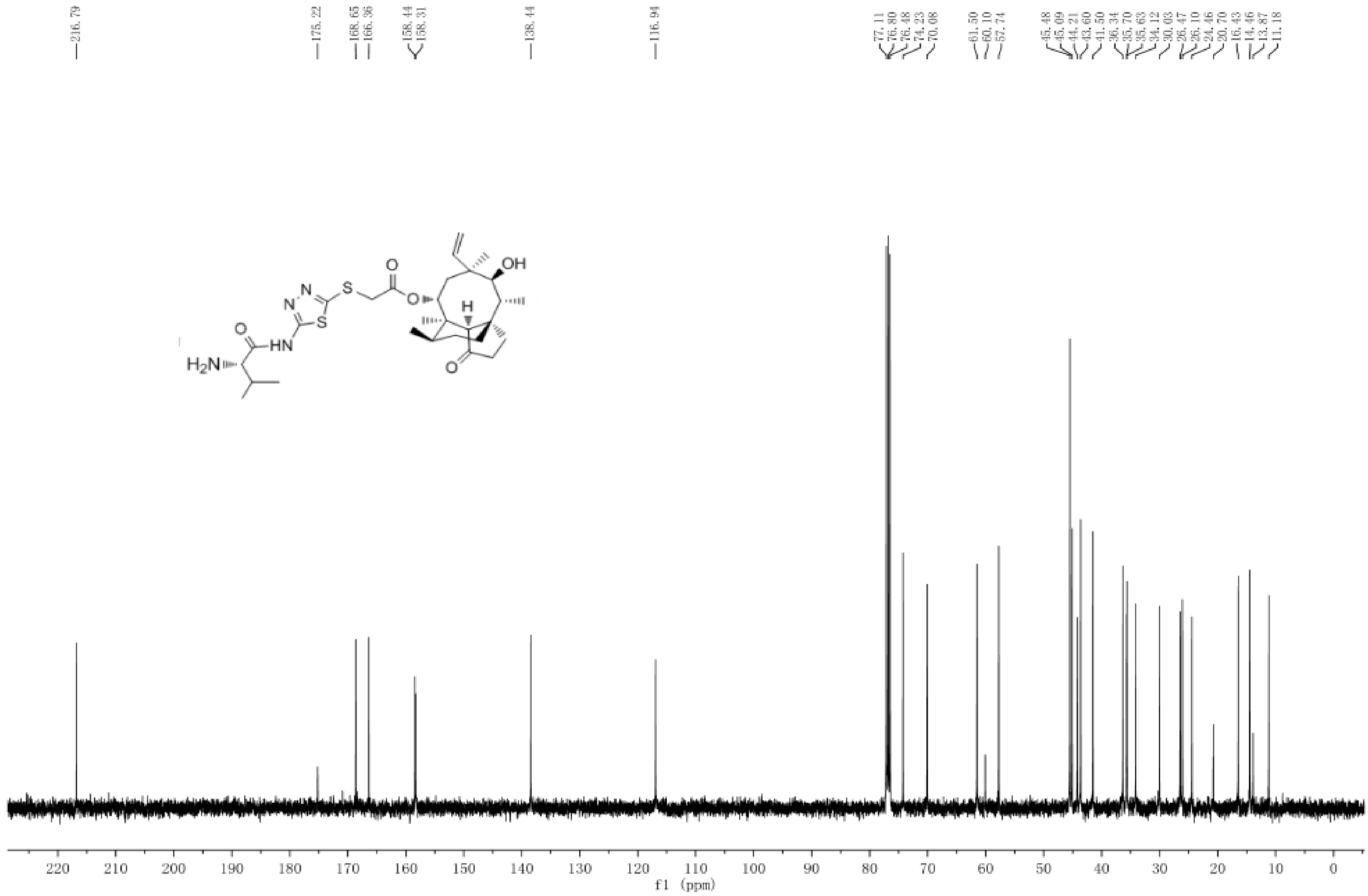

**Figure S25.** IR spectrum of compound 6c.

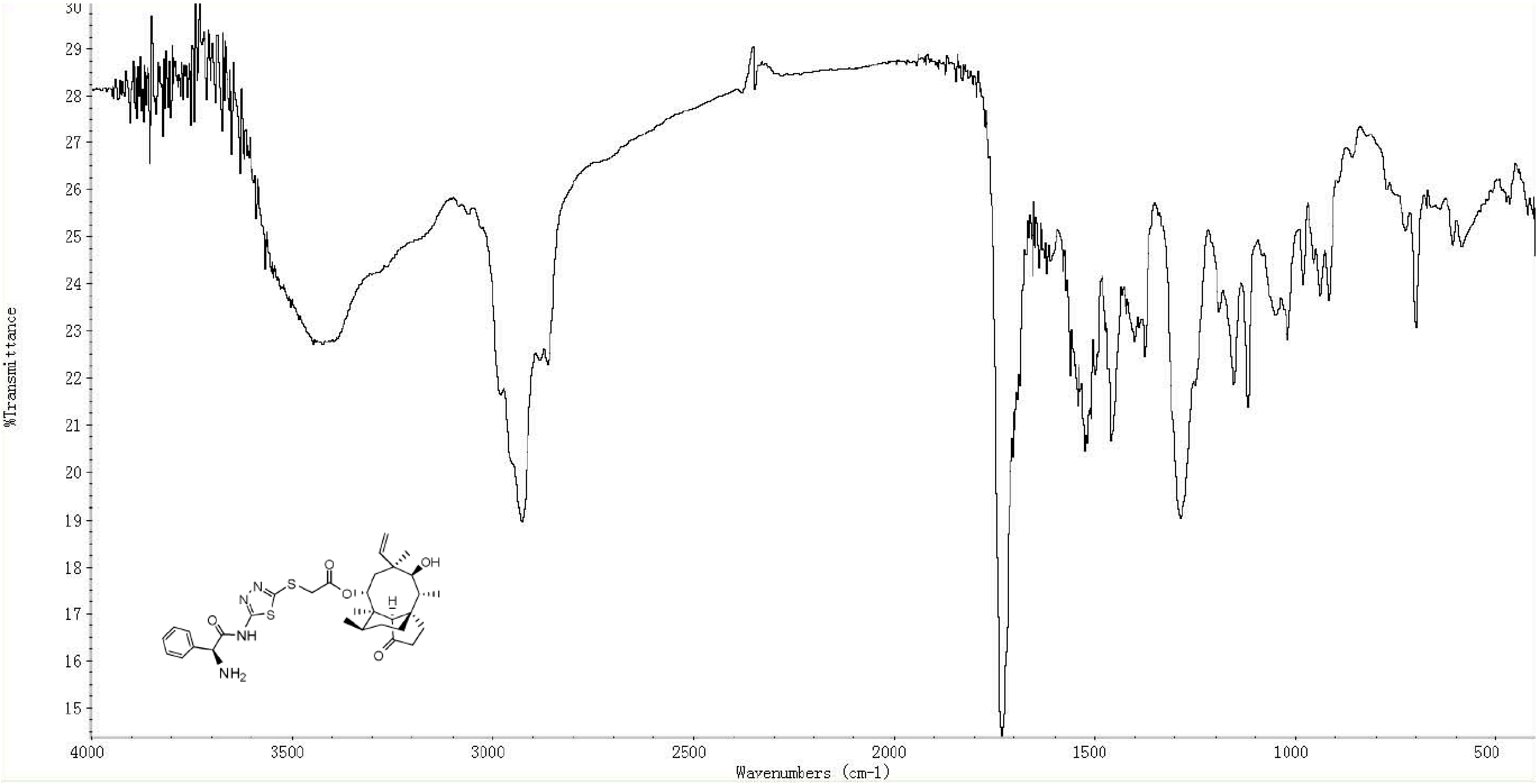

**Figure S26.** $^{1}$H NMR spectrum of compound 6c.

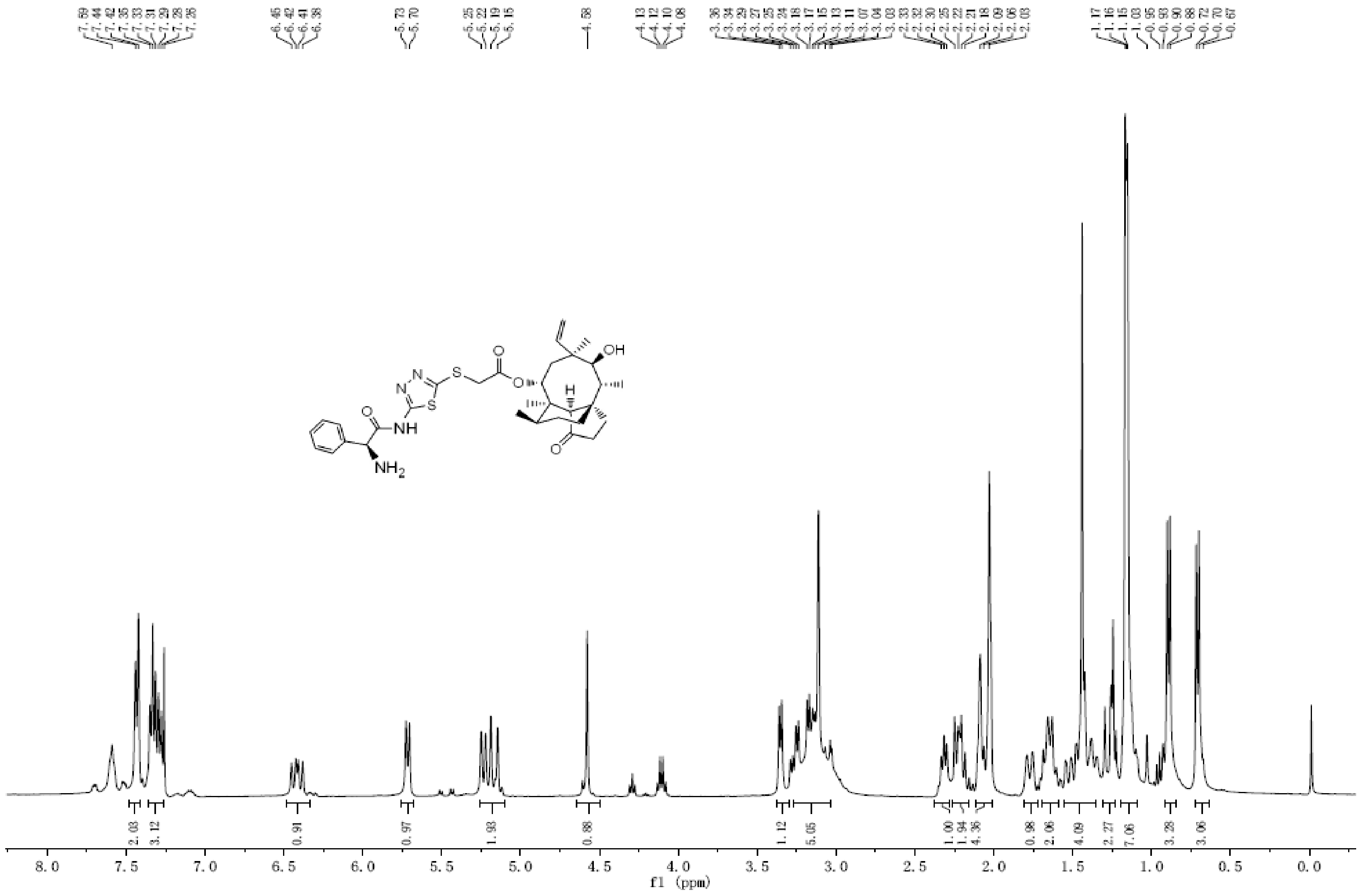

**Figure S27.** $^{13}$C NMR spectrum of compound 6c.

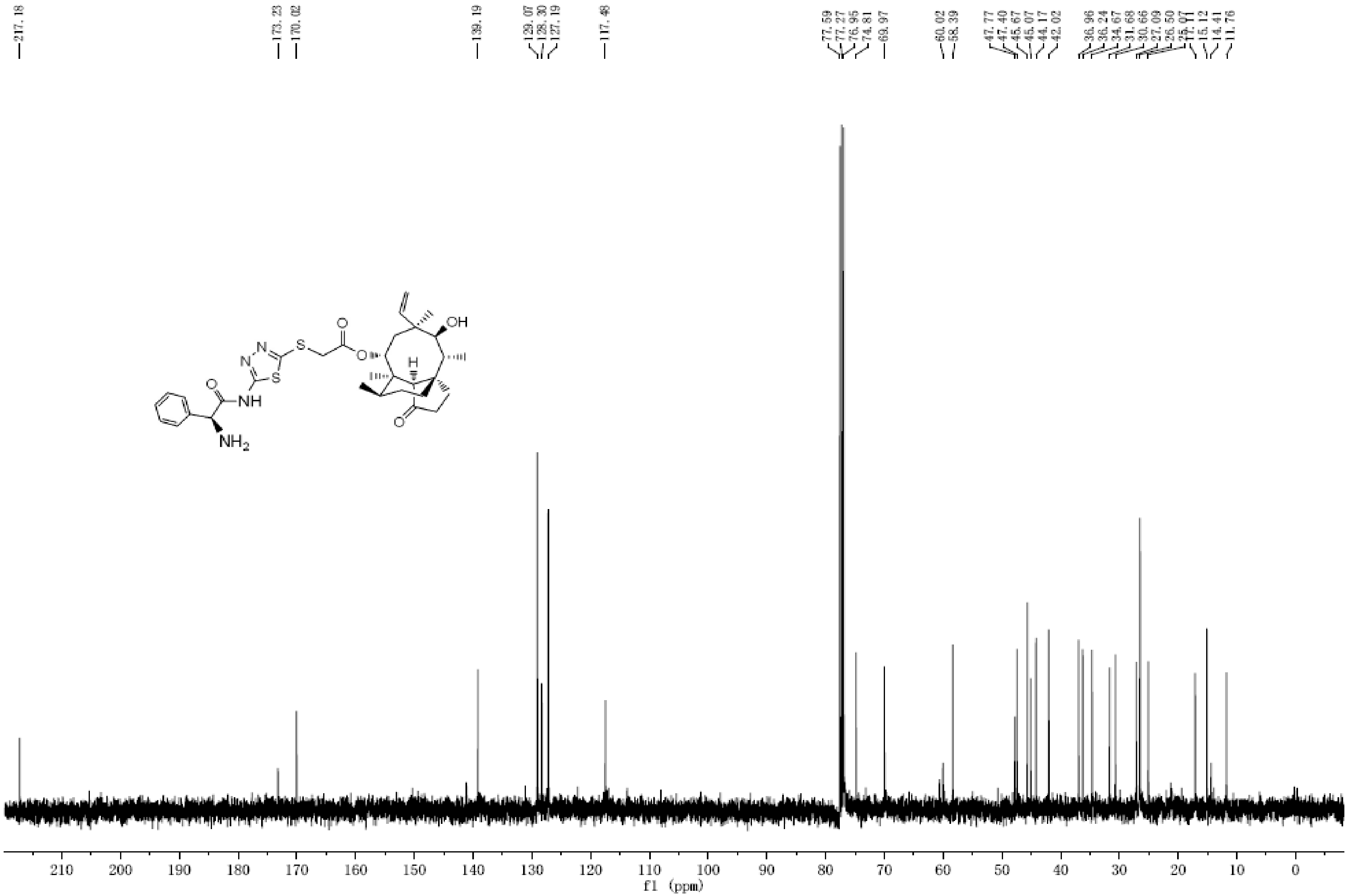

**Figure S28.** IR spectrum of compound 6d.

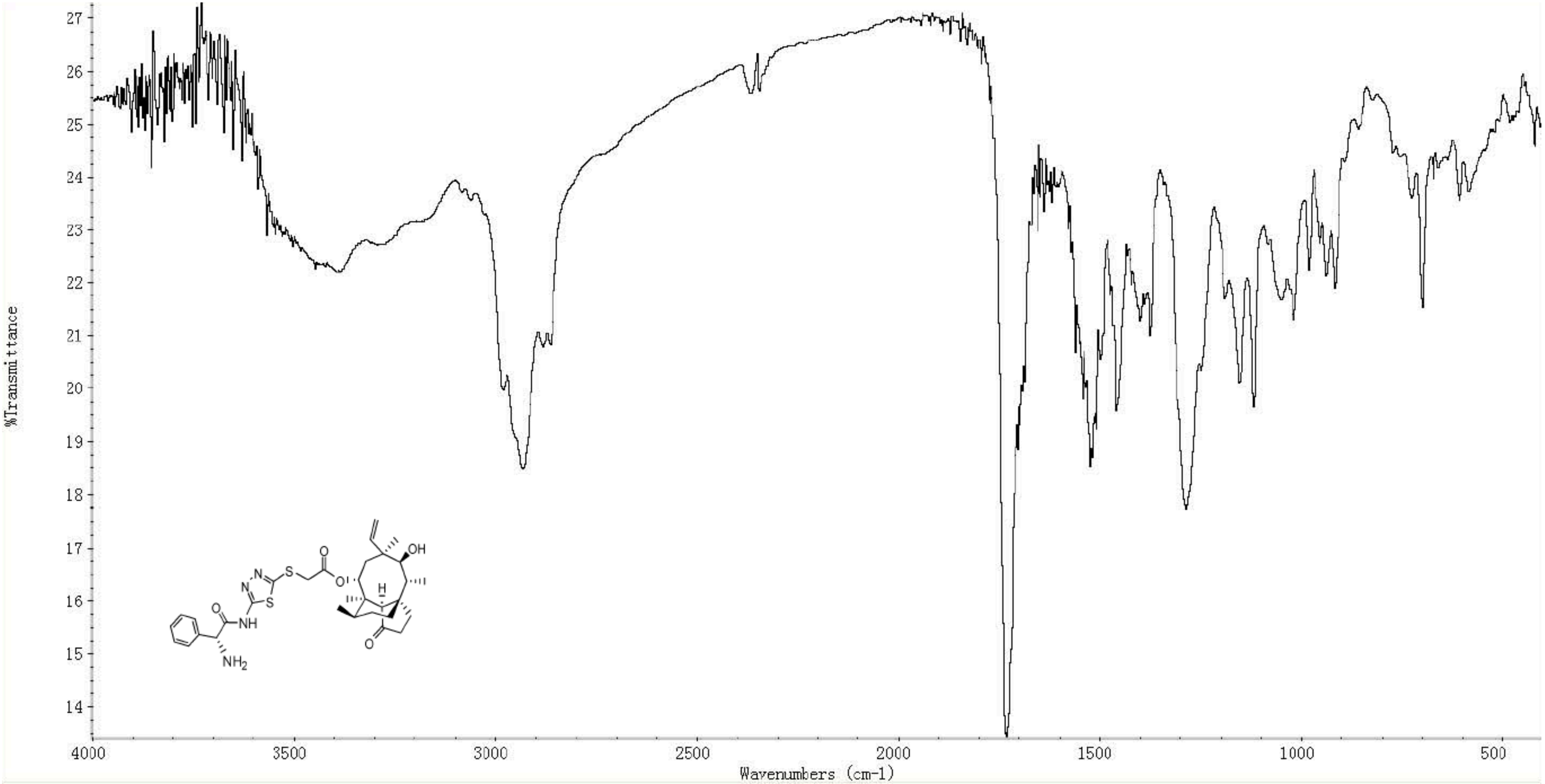

**Figure S29.** $^{1}$H NMR spectrum of compound 6d.

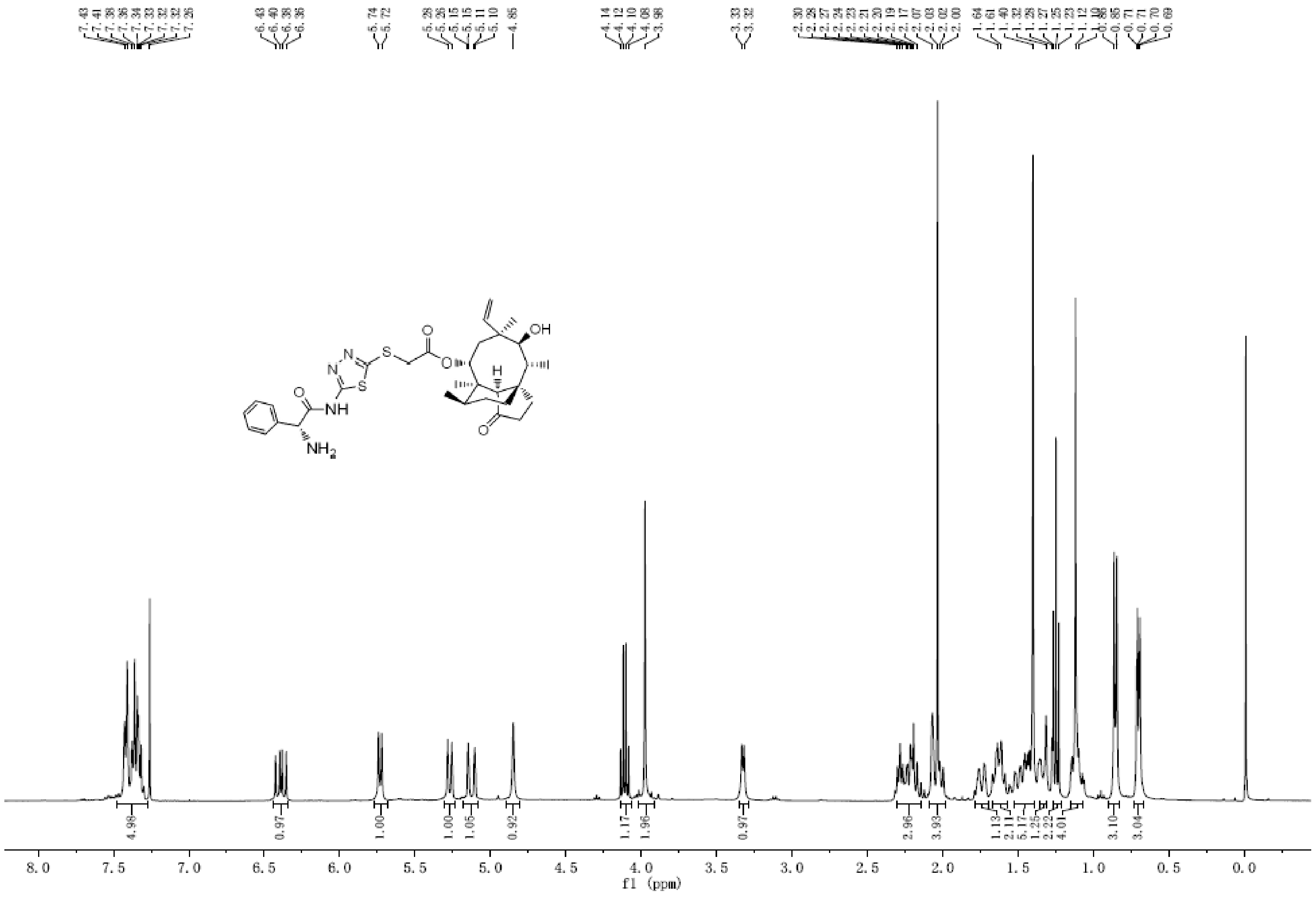

**Figure S30.** $^{13}C$ NMR spectrum of compound 6d.

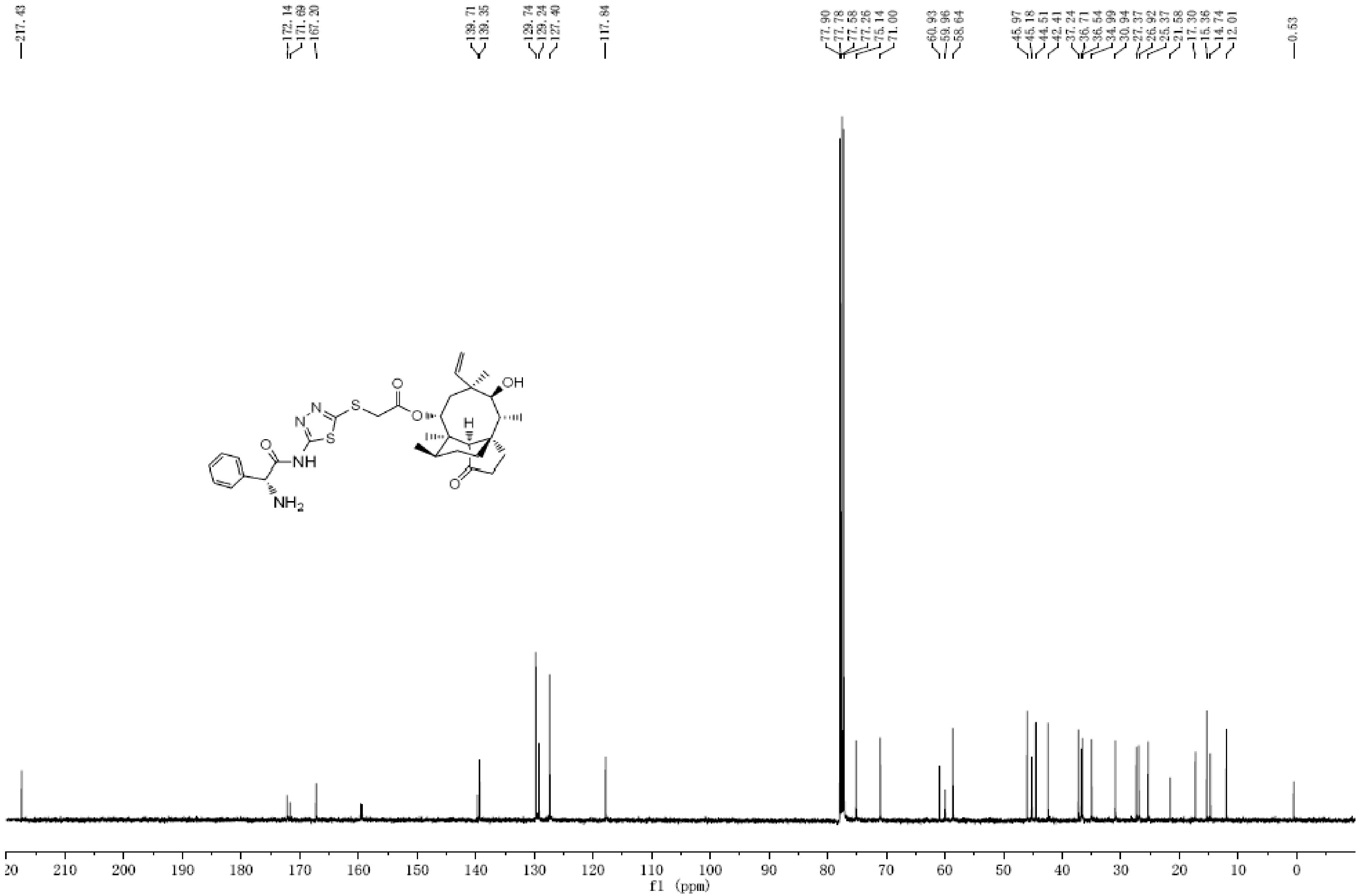

Supplement: Supplementary file 1 [file molecules-19-19050-s001.pdf]
